# Models, outcomes, barriers, and facilitators of supportive care in cancer: a scoping review

**DOI:** 10.1007/s00520-026-10613-1

**Published:** 2026-03-27

**Authors:** Daniel Monnery, Kate Law, Dipesh P. Gopal, Ollie Minton, Lynn Calman, Charlotte Chamberlain, Sally Taylor, Roger Smith, Tomoko Lewis, Avril Chester, Stephanie Lister-Flynn, Joanne Droney

**Affiliations:** 1 Faculty of Health and Life Sciences, University of Liverpool, Liverpool, UK; 2 Department of Palliative and Supportive Care, The Clatterbridge Cancer Centre NHS Foundation Trust, Wirral, UK; 3 The Christie Patient Centred Research Team, University of Manchester, Manchester, UK; 4 Wolfson Institute of Population Health, Queen Mary University of London, London, UK; 5 Department of Palliative Medicine, Royal Sussex County Hospital, Brighton, UK; 6 Centre for Psychosocial Research in Cancer, University of Southampton, Southampton, UK; 7 Palliative and End of Life Care Research Group, University of Bristol, Bristol, UK; 8 Department of Palliative and Supportive Care, Somerset NHS Foundation Trust, Somerset, UK; 9 Royal Preston Hospital, Lancashire Teaching Hospitals NHS Foundation Trust, Preston, UK; 10 London, UK; 11 University Hospitals Sussex NHS Foundation Trust, Worthing, UK; 12 The Symptom Control and Palliative Care Team, The Royal Marsden NHS Foundation Trust, London, UK

**Keywords:** Enhanced supportive care, Models, Outcomes, Facilitators, Barriers

## Abstract

**Purpose:**

There is wide heterogeneity of supportive cancer care provision in the UK. Greater understanding of evidence-based models is key to developing standards for care. The purpose of this scoping review is to identify the evidence-based models of delivering supportive care to patients with cancer, the outcomes measured, and the facilitators and barriers to accessing supportive care.

**Methods:**

We conducted an extensive search using MEDLINE, SCOPUS, PsychINFO, EMBASE, EMCARE, CINAHL from 2000 until 2025 to identify (i) the existing service and workforce models/designs supporting supportive care delivery; (ii) the benefits, costs, and outcomes relating to these services; and (iii) the facilitators and barriers to setting up services. Data were analysed using tabular summaries and content analysis.

**Results:**

One hundred and fifteen articles were analysed. Thirty-six distinct models of supportive care delivered by different professional groups were identified. Outpatient multiprofessional clinic models demonstrated the greatest number of positive patient outcomes. Positive outcomes were also described from digital, educational, and patient navigation models. Quality of life was the commonest reported primary outcome. Facilitators and barriers were described within five overarching themes: knowledge and understanding among healthcare professionals, clinical resource, logistics and organisation, patient-specific considerations, and digital considerations.

**Conclusion:**

A variety of evidence-based models were identified with a range of outcome measures and a plurality of described facilitators and barriers. Future work involving patients and professionals delivering existing supportive care services is needed to investigate which models could be adopted at scale within the NHS to facilitate the standardisation of supportive care in UK cancer care.

**Supplementary Information:**

The online version contains supplementary material available at 10.1007/s00520-026-10613-1.

## Introduction

Supportive care is the holistic management of symptoms and side-effects related to a cancer and its treatment, from cancer diagnosis right through to survivorship and end-of-life care [1]. The development of supportive care is part of a wider movement nationally [2] and internationally to more person-centred holistic care beyond the scope of treating the cancer alone [3].

In the UK, enhanced supportive care (ESC) is a comprehensive multi-disciplinary approach which supports the provision of proactive, holistic and supportive care for patients and those important to them, delivered early in the disease trajectory, often alongside anti-cancer treatment [4]. ESC as a model of cancer care was initially launched as a national quality improvement initiative in 2016 [5]. Today, ESC services provide timely, multiprofessional, coordinated care to patients in approximately 25 cancer centres in England [4].

ESC services are currently not nationally commissioned in the UK and therefore whilst available in some centres as extensions of existing services, are not available in all cancer services. There is no standard framework to guide service delivery or implementation. ESC services have developed iteratively to support local needs, leading to heterogeneity in scope, context, settings and delivery [6]; however, the core components and models of ESC services (those which should be present within all models) have not been defined. Although there are data to show that ESC is associated with improved symptom control, quality of life and reduced healthcare costs [7, 8], the “essential ingredients” of ESC are unclear, and the comparative impact of ESC, compared with other aspects of cancer care, is unknown [6]. Factors supporting ESC engagement and implementation have also not been characterised.

A recent commissioned review of palliative and end-of-life care in the UK highlighted the benefits of ESC for the sustainability of the national health service [9] but acknowledged the lack of systematic and equitable adoption [10]. A better understanding of the effective components of ESC is needed to support the evidence-based development, funding, and implementation of equitable services. It will also identify priority areas for research in this growing field.

A rapid review of the literature revealed the widespread use of the terminology “supportive care” rather than “ESC”. Since ESC is an emerging clinical concept and primarily UK-based at present, and since the term ESC is not universally adopted, we hypothesise that a better understanding of approaches to supportive care delivery will provide much needed evidence to inform the ongoing development and implementation of ESC.

This aim of this scoping review is to describe the breadth of evidence within the international literature concerning supportive cancer care models, the benefits, costs and outcomes, and the key facilitators and barriers to their implementation.

## Methods

A scoping review was preferred over systematic review methodology for this study, to identify the breadth of existing heterogenous research and the gaps in research literature [11]. The methodology is based on the framework by Arksey and O’Malley [12] and guidance from the Joanna Briggs Institute [13, 14]. The five stages of the review included identifying the research question and relevant studies; study selection; charting the data; and collating, summarising, and reporting the results.

A team-based approach was used in applying the scoping methodology [15]. This study was designed, developed and delivered in collaboration with the UKASCC Enhanced Supportive Care National Collaborative [16]. Multiprofessional stakeholders from clinical, academic and commissioning backgrounds, as well as patient and public contributors, developed the research question and research protocol and interpreted the results.

Findings were reported according to Preferred Reporting Items of Systematic Reviews and Meta-analyses extension for Scoping Reviews (PRISMA-ScR) standards [17]. The scoping review protocol was registered with the Open Science Framework on 2023-09−29: https://osf.io/zxpju/.

The Population, Concept, Context approach [14, 18] was used to develop and refine the research questions and define the inclusion/exclusion criteria:

- Population: adults with cancer diagnosis.
- Concept: supportive care.
- Context: English language, primary and secondary quantitative and qualitative research, as well as reports from the grey literature.

Inclusion and exclusion criteria are shown in Supplementary Figure 1.

This scoping review is aimed at answering the following research questions:

1. What are the existing service and workforce models/designs supporting the delivery of (enhanced) supportive care in cancer?
2. What are the benefits, costs, and outcomes relating to the delivery of (enhanced) supportive care in cancer?
3. What are the facilitators and barriers to setting up/delivering (enhanced) supportive care services/interventions?

The following databases were searched for eligible papers from January 1, 2000, until 24/02/2023: MEDLINE, SCOPUS, PsychINFO, EMBASE, EMCARE, and CINAHL. The search strategy is shown in Supplementary Figure 1. Forward and backward citation ensured inclusion of all relevant studies.

All studies were downloaded from their respective databases, removing any duplicate records. The remaining records were uploaded to an online systematic review platform “Rayyan” [19], and records were independently screened for inclusion at abstract and full text stages by teams of two researchers (DM&JD, RS&DG, OM&KL). Any conflicts were resolved through consensus between screening researchers and an independent researcher from one of the other teams if required. The full text of the remaining records was independently screened by teams of 2 researchers (DM&JD, OM&KL, and DG&RS). Additional articles captured through grey-literature search during the full text review of included articles were independently screened by teams of two researchers (DM&ST, JD&TL, KL&LC, OM&CC, and DG&RS).

A repeat search, using the same terms, databases, and process, was conducted in March 2025 to account for any new articles published during the period of initial data extraction and synthesis. These articles were screened by authors DM and JD.

### Data extraction

The results were extracted into a review-specific template which was refined iteratively [13]. The final template included the following categories: author’s name, year, country, region or location, a brief description of the supportive care model, components of the model, length of time of model, number of patients involved, cancer type, cancer staging, time of follow-up, measured outcomes, validation of measured outcome, facilitators and barriers, and study design. Data extraction, mapping and charting was carried out independently in teams of two researchers as above and verified with back-forth dialogue to ensure there was 100% agreement. Provision was made for any disagreements to be resolved by a third researcher; however, this was not needed. In line with guidance for scoping reviews, quality assessment was not undertaken [20].

Data synthesis was carried out in working groups with each group focusing on one research question (Models (DM/KL/TL), Benefits, Costs and Outcomes (DM/JD) and Facilitators/Barriers (OM/SL/JD) [21]. Data synthesis included both quantitative and qualitative analyses. Quantitative data were described using counts and percentages. Qualitative data were analysed using thematic analysis [22]. Codes were checked and preliminary themes verified within the working groups. Models of care were described in terms of professional groups involved and components of care and interventions offered. The patient and health-service-related outcomes were categorised and the associations classified as either positive (beneficial) or negative (no benefit demonstrated). An inductive approach was applied to the categorisation of barriers and facilitators into themes [23].

## Results

**Fig. 1:**
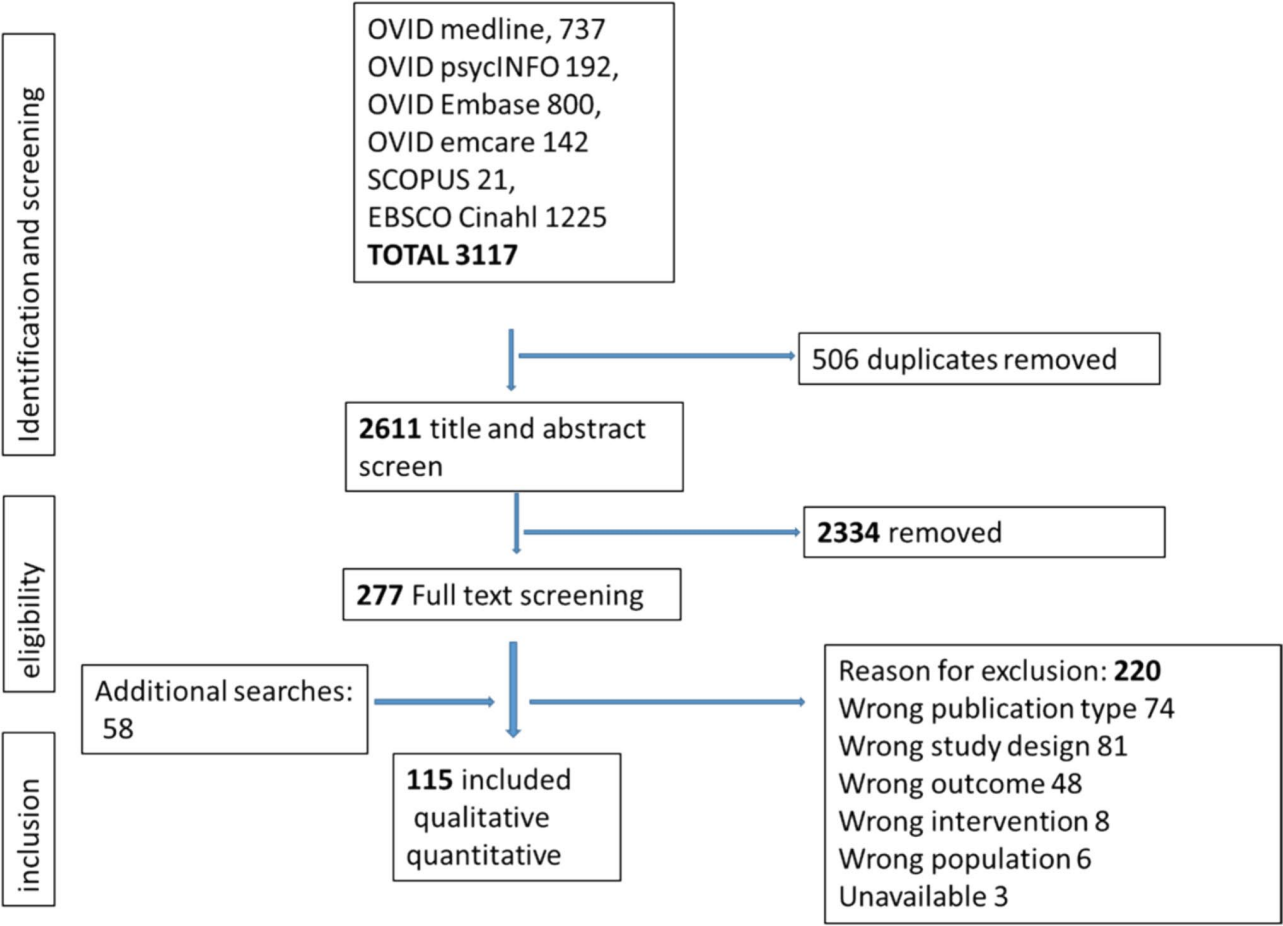
Preferred reporting items for systematic reviews and meta-analysis flow diagram detailing study selection for inclusion in this review

Of 3117 articles initially captured through the searches and 58 articles added through hand searching, 115 studies met the inclusion criteria and data were extracted for the final analysis (Fig. 1). The descriptive characteristics of included studies are presented in Table 1 with further detail in Supplementary Table 1.

**Table 1:** Characteristics of included articles

**Study design**	*n* (%)
RCT	45 (39)
Non-randomised intervention studies with control arm	7 (6)
Prospective pre-post-test single cohort intervention study	5 (4)
Prospective single cohort study	20 (17.4)
Prospective cross-sectional study	3 (2.6)
Retrospective cohort study with comparison group	6 (5)
Retrospective single cohort study	11 (9.6)
Reviews	3 (2.6)
Surveys	3 (2.6)
Qualitative studies	11 (9.6)
Mixed methods	14 (12)
Cancer diagnosis of participants	
Breast	34 (29.6)
Genito-Urinary including prostate	24 (20.9)
Lung	20 (17.4)
Gynaecological	15 (13)
Head and Neck	12 (10.4)
Gastrointestinal	23 (20)
Haematological	8 (7)
Melanoma	5 (4.3)
Sarcoma	2 (1.7)
Mixed tumour types	47 (40.9)
Stage of disease	
Early stage (stage 0-II or amendable to survey)	17 (14.7)
Advanced disease (locally advanced, stage 4, recurrent disease, patients at end of life)	28 (24.3)
All stages of cancer included	37 (32.1)
Stage of disease not specified	38 (33)
Year of publication	
Prior to 2010	1 (1)
2010–2020 inclusive	59 (51)
2021 to March 2025	55 (48)

Within the final articles, 45 were randomised controlled trials (RCTs), seven were prospective non-randomised intervention studies and five prospective pre-post-test intervention studies. We included 23 prospective studies without a direct comparator cohort, one of which used national data as a reference cohort. We included 17 retrospective studies, six of which had a comparator cohort. 11 studies were qualitative in design. 14 studies used mixed methodologies. There were three surveys and three review articles.

**Fig. 2:**
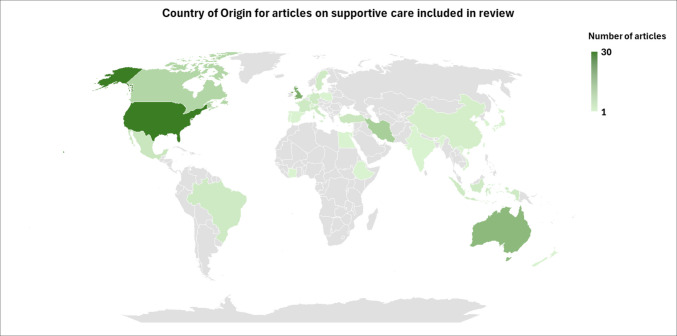
World map showing the country of origin for articles on supportive care models, outcomes, facilitators and barriers included in this review

One-third of articles included participants from North America, with 28% and 20% from Europe and Asia respectively (Fig. 2). The most common cancer diagnosis was breast (*n* = 34). Disease stage ranged from early stage (stages 0–2 or amendable to surgery, 17 papers) to advanced disease (locally advanced, stage 4, recurrent disease, or patients at end of life, 28 papers), with 37 studies including patients with all stages and 33 papers in which stage of disease was not specified.

Many studies used mixed participant populations; therefore sub-totals > 115.

Ninety-four articles described models of care, 63 described outcomes and 53 described facilitators and barriers. Most articles contained data related to more than one area and had data extracted for each.

### Findings from studies describing models of care:

**Fig. 3:**
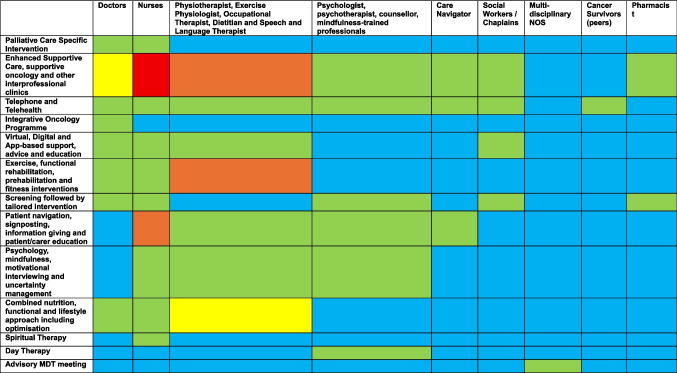
Heatmap of frequency of studies linking different professional groups to models of care. Key: 0 studies (blue), 1–5 studies (green), 6–10 studies (yellow), 11–15 studies (orange), and  > 15 studies (red)

Of the 94 studies describing models of supportive care, 36 distinct models were described (Supplementary Table 2). Models were described based on the disciplines of professionals involved, the setting of delivery, the mode by which they interfaced with patients (e.g. face to face, telehealth, digital) and, where possible, the phase of illness in which they provided patient care. Ten studies reported models of supportive care within hospitals [24–33], 36 clinic-based [7, 34–68] and 22 community-based [69–90]. The remainder (22) either described combinations of settings or the setting was unknown. Figure 3 (and Supplementary Table 2) summarises the healthcare professional groups associated with the provision of different models of supportive care.

### Professional groups involved in the delivery of care

Doctors from palliative care, oncology and other medical specialties tended to be involved in outpatient clinic-based models including supportive care/supportive oncology clinics [49, 56, 57], enhanced supportive care [58, 59, 68] and early palliative care [59]. Doctors were also associated with inpatient supportive and palliative care including integrated geriatric/palliative care models [24]. Nurses were associated with a greater diversity of clinical models. Whilst nurses were frequently involved in the same outpatient supportive care models as doctors (Fig. 3), nurses were also associated with patient navigation [36], education [78, 80, 91–93], group-based [33], screening-based [44, 50, 61, 94], and information giving models [33, 95]. Nurses were also the most common professional group involved in the studies offering most care through digital tools [29, 70, 71, 85, 86], and telehealth [63, 96]. Allied health professionals (physiotherapists, occupational therapists, dietitians, social workers, and pharmacists) were predominantly involved in the delivery of multiprofessional supportive care/supportive oncology [49, 53, 57, 60, 97], enhanced supportive care [8, 59, 68], and patient navigation services [98]. Each of these professionals were involved in services specifically relating to prehabilitation [81], rehabilitation [99], and lifestyle interventions [39, 45, 100] to a greater extent than any other group. A similar pattern was seen with psycho-oncology professionals including psychologists, psychotherapists and counsellors who, as well as being involved in the delivery of multiprofessional supportive oncology [49] and enhanced supportive care services [58,, also delivered bespoke interventions relevant to their scope of practice including cognitive behavioural therapy [90], psychotherapy [101], and motivational interviewing [45].

Supportive care/supportive oncology clinics and models which described themselves as enhanced supportive care represented multiprofessional models with the greatest documented diversity of professionals involved (Fig. 3). Inpatient supportive oncology services were notably less diverse, with only doctors and nurses being involved in studies describing this model of care [24]. Furthermore, fewer studies reporting digital interventions included multiprofessional delivery compared to clinic models (Fig. 3).

There was only one study describing the role of cancer survivors in the delivery of supportive care interventions [96]. Models which included speech and language therapists as part of the intervention were less common, with only one study describing the role of this professional group [54].

### Identification of individual patient’s needs

Two studies reported models of screening-based access to multidisciplinary support for patients. Whilst neither study showed any improvement in overall quality of life, Geerse et al. reported that as a result of their intervention, fewer patients in the experimental group received chemotherapy in the last weeks of life [102] and Ke et al. reported higher physical functioning, role functioning, and activity levels at nine months and lower psychological distress at 12 months [44]. One article reported on the outcome of screening-based personalised care for caregivers [94]. This study showed the delivery of tailored support based on routine screening of needs resulted in family caregivers feeling better prepared in caregiving than controls [94]. Furthermore, the proportion of family caregivers in the intervention group with high distress significantly decreased during the study as well as the number of problems they reported [94]. In addition, the use of screening tools and patient-reported outcome measures (PROMs) were recognised within this review as supporting patient-centred discussions and care [36, 56, 61, 62].

### Education-based interventions

Seven studies included in this review reported outcomes from educational interventions. Three studies, all reporting digital app or web-based models of education, described outcomes including improvements in self-esteem and anxiety [86], high patient satisfaction [86], better global quality of life [73, 85], improved emotional function [85], and decreased number of supportive care needs [73]. A further three studies reporting other models of educational interventions described outcomes including high patient satisfaction [103], improvements in physical health, role, emotions, cognition, social function and global quality of life [31], and improvements in symptoms and global symptom distress [104]. One study reporting on the impact of a group combined exercise and education intervention showed no significant difference in anxiety or depressive symptoms compared to usual care [105].

### Findings from studies describing benefits, costs, and outcomes

**Fig. 4:**
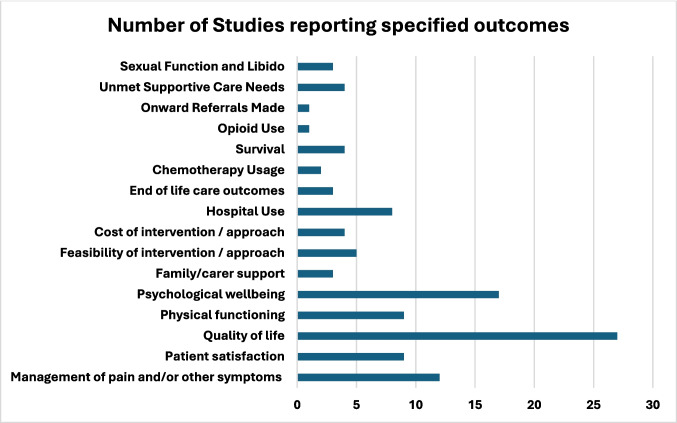
Frequency of outcomes examined within included studies

Sixty-three studies reported on the patient-centred/health-service related outcomes, many of which reported on more than one outcome. Figure 4 describes the frequency of outcome measures within the included studies. Twelve studies reported on pain and symptom control as an outcome [7, 8, 25, 37, 57, 75, 78, 87, 90, 106–108], nine on patient satisfaction [35, 36, 103, 106, 107, 109–112], 27 on quality of life [31, 32, 37, 42, 44, 47, 48, 57, 64, 65, 68, 70, 73, 75, 78, 85, 92, 100, 102, 103, 105, 107, 108, 113–116], nine on physical functioning [44, 71, 85, 90, 112, 117–120], 17 on psychological wellbeing [25, 30, 37, 42, 57, 69, 71, 78, 86, 92, 94, 101, 103–106, 114], three on family/carer support [33, 91, 106], five on feasibility [47, 62, 88, 117, 118], four on cost [8, 24, 99, 121], eight on hospital use [8, 34, 56, 57, 60, 65, 121, 122], three on end of life care outcomes [56, 57, 65], two on chemotherapy usage [7, 102], four on survival [7, 34, 47, 68], one on opioid use [122], one on onward referrals made [122], four on unmet supportive care needs [33, 70, 73, 92], and three on sexual function and libido [91, 96, 113].

Fifty-one studies reported positive findings, with an additional 11 reporting mixed outcomes. Interventions described in studies reporting improvements in quality of life included exercise interventions [100, 115], mindfulness [64], cognitive behavioural therapy and hypnosis [42], group-based education [31], web-based education [73, 85], nurse-led supportive care programmes [48], integrative oncology [108], psychosexual counselling [113], enhanced supportive care [68], supportive care/supportive oncology outpatient clinics [57], and early palliative care [107].

Studies which reported improvements in pain and symptom control included exercise [87], integrative oncology [108], enhanced supportive care [7, 68, 106], supportive care/supportive oncology clinics [57], early palliative care [107], hospital palliative care [25], and digital cancer survivorship resources [75].

Interventions which improved psychological wellbeing including positive outcomes for systematic distress screening and tailored support [94], Telephone Interpersonal Counselling (TIPC) and Supportive Health Education (SHE) [104], integrative oncology [30], interpersonal psychotherapy [101], digitally accessible psycho-education [86], support [71], and lifestyle coaching [69], cognitive behavioural therapy and hypnosis [42], enhanced supportive care [106], and hospital palliative care [25].

Interventions which demonstrated cost-effectiveness included cardiac rehabilitation (following cardiac sequelae from anticancer treatment) which demonstrated a favourable incremental cost-effectiveness ratio using quality adjusted life years compared with usual care [99] and supportive care/supportive oncology services which included inpatient [24], ambulatory [121] and enhanced supportive care models [8] with varying degrees of savings reported. Dedicated supportive care/supportive oncology services reported a reduction in hospital admissions ranging from 3.2% reductions (and a 2.2% cost reduction) from a day case model [121] and an 8–31% reduction from supportive oncology clinic models [56, 60]. There was also a positive reduction in hospital admissions following the use of an inpatient palliative and supportive care unit [122] and from outpatient enhanced supportive care which showed 1472 avoided admissions for a population of 4594 patients, leading to a cost saving of five times the cost of the service [8]. Furthermore, the admission reduction resulting from an inpatient supportive care service in one study resulted in a 1.4 benefit cost ratio [24]. Only one small study measured hospital use as an outcome of a community-based intervention (no reduction) [80].

Multiprofessional outpatient clinics were the only models associated with improved survival including enhanced supportive care [68] and interprofessional disease-specific clinics [47].

Only one study reported only negative findings in terms of no difference in hospital use/aggressiveness of end-of-life treatments between patients seeing the integrated outpatient-based multi-disciplinary supportive care team and the control group [65].

### Findings from studies describing facilitators and barriers to setting up/delivering supportive care services/interventions

A description of facilitators and/or barriers to the implementation or delivery of supportive care interventions was provided in 53 studies. These were described within five themes: (1) knowledge and understanding among healthcare professionals, (2) clinical resources, (3) logistics and organisation, (4) patient-specific considerations, and (5) digital considerations. These are summarised in Table 2.

**Table 2:** Facilitators and Barriers to the delivery of/implementation of supportive care models and interventions

Barriers	Facilitators
Knowledge and understanding among healthcare professionals	
Lack of knowledge about the intervention or services available	Standardising timing of referral/intervention, duration and scope of intervention
Lack of understanding about impact of services or intervention	Engagement from both managers and clinicians
Overlap in roles and responsibilities	Providing training
	Collaboration between teams
Clinical resources	
Lack of staffing or time	Dedicated staff to run service/intervention
	Sharing rather than duplicating resources
Logistics and organisation	
Difficulties identifying eligible patients	Proactive identification of eligible patients
Appointment scheduling conflicts	Flexibility in clinic scheduling and setting (free-standing or embedded clinic, inpatient consultation)
Rigid timetabling of contact and intervention	
Geographical location of clinic	Flexibility in mode of communication i.e. face to face/telephone/email/ePROMS
Patient-specific considerations	
Level of patient need: • Physically too unwell to engage with intervention • Disease progression impacting duration and effectiveness of intervention • Patients with significant existing needs may be already known to support services • Cultural barriers impacting health seeking behaviours/willingness to disclose need	Not "One size fits all"
	Making information about the service available for all patients
	Provision of a holistic approach, tailored to the needs of the individual
	Reframing models of care to be culturally sensitive and cognisant of different methods of coping/health seeking behaviours
	Using personal needs assessments to demonstrate needs
Reduced adherence to the intervention if no supervision	Proximity to the location of the service/intervention
Financial resources: travel costs, parking	Financial support for transport/parking
Competing patient priorities including time	Patient feedback
Location and mobility	Involving family/caregivers
Digital considerations	
Technical challenges i.e. performance of the intervention/app	Caregiver can provide support for access to digital intervention/app
Levels of literacy, digital literacy, visual acuity, digital skills	Telehealth to reduce waiting lists and enable more family members to join consultation
Concerns about confidentiality	
Impact on communication and preferences for face-to-face visits	
Burden of some digital interventions	

### Knowledge and understanding among healthcare professionals

A clear understanding of the service or intervention was identified as a critical facilitator to the successful delivery and implementation. For patients, awareness and understanding were fostered by proactive information provision, rather than waiting for patients to request information [123]. Providing information in a simple and accessible manner, avoiding medical jargon, was important [82, 124]. Clinicians’ knowledge and understanding determined whether or not they engaged with or prioritised the service/intervention [56, 123, 125, 126]. A lack of clear communication about the components of the intervention/service, eligibility criteria, and aims can result in misinterpretation and misunderstanding [94]. Defining the roles of clinical team members and standardising referral was recognised facilitators in implementation [36, 56, 95, 127]. Late or delayed referral hindered the effect of the intervention [46, 106]. Just as a lack of integration with existing services was identified as a barrier [95, 128], authentic integration promotes engagement [52, 53]. Knowledge of the service/intervention enabled clinicians and patients to appreciate the need and benefits [54]. When there was a highly perceived necessity of the service by patients, carers, and clinicians, there was a low rate of patient non-attendance [54]. Clinicians also identified additional benefits, such as improved clinical flow, which facilitated adoption and engagement [66]. Engagement from both clinicians and management was identified as beneficial [35].

### Clinical resources

Resources (time and staffing) were identified as both facilitators [56, 125] and, if lacking, barriers to service/intervention delivery [6, 33, 39, 52, 54, 62, 97, 125]. Resource allocation and management, including sharing MDT expertise, avoidance of unnecessary clinical visits, and careful clinic/intervention scheduling, supported clinician engagement [56]. Having resources specific to the service/intervention was a facilitator, such as a dedicated palliative care service/staffing [34, 56, 62, 65].

### Logistics and organisation

Organisation and scheduling played an important role. Schedules that were too rigid or fixed presented barriers to engagement and resulted in missed appointments [28, 36, 94]. Many sources referred to the negative impact of environmental factors such as geographical location and resulting transport issues, as well as the scheduling time of the day [66, 91, 107, 108, 125, 127–129]. Transport and parking provision mitigated some of these barriers [123], as did flexibility in scheduling, mode of consultation (face to face versus virtual/telephone), and setting [29, 53, 96, 98, 123, 125].

### Patient-specific considerations

A key facilitator was being able to tailor the intervention/service to the specific needs or circumstances of the individual patient [65, 108, 123]. For some patients, this was about recognising gender differences in health engagement [74, 124, 126]. For others, it was about language [44, 107], or adopting culturally sensitive approaches [74, 104, 107, 108]. Proactive engagement with patients was also a facilitator [52, 123, 124]. Patient Reported Outcome Measures and the use of screening or needs assessment tools were recognised as supporting patient-centred discussions and care [36, 56, 61, 62].

Financial burden was cited by a number of papers as a barrier to engagement [110, 123, 124]. Some patients were too unwell to participate in a new service/intervention [96, 115, 129]. Other patients, because they were so unwell or because their needs were already identified, were already known to a supportive care service [65], limiting the impact of a new intervention.

### Digital considerations

Although digital literacy and technical problems were identified as potential barriers [27–29, 70, 79, 98, 111, 123], the benefits of digital/App-based delivery mechanisms were reported to enhance accessibility for some patients [76]. The capacity for adoption of technology was demonstrated [75], and digital facilitators such as family/carer/staff support were identified [70, 76].

Some papers reported on the verbal/non-verbal communication barriers posed by tele-health interventions [29] or concerns about confidentiality [125]. Others reported on the benefits of telehealth on access and engagement with family members [29, 96, 98].

## Discussion

The Multinational Association of Supportive Care in Cancer (MASCC) and American Society for Clinical Oncology (ASCO) have published the MASCC‑ASCO standards and practice recommendations on survivorship care for people affected by advanced or metastatic cancer [130]. The results from this scoping review add to this work in the description of the evidence around the models, benefits and outcomes of supportive care, as well as factors influencing implementation.

### Supportive care: one size does not fit all

The most immediate finding from this review is the profound heterogeneity in models of care, outcomes measured for the diverse patient demographics and settings in which supportive care has been implemented. Supportive care is provided across healthcare settings: in hospital, in the clinic and in the community for patients with a variety of cancer diagnoses, at different stages of their cancer journey, from early diagnosis, through treatment, to survivorship and end of life care. Interventions ranged from multi-professional clinical services to speciality-specific approaches such as physical therapy, psychological support etc. The data from this review provides evidence that supportive care is a complex intervention, with multiple interacting and changing components relating to the patient, the disease, the treatment and the health service set up [131, 132]. Evaluation of barriers and facilitators demonstrated the need to tailor the models and delivery of services/interventions according to individual patients’ needs and local system factors. The multi-modal approaches and multi-disciplinary teams included in the studies within this review point to the complexities of needs faced by patients with cancer, across all diagnoses and stage of disease [132–134]. It also highlights the appropriate tailoring of services to the context in which they operate. However, without consistency in models, greater attempts should be made to align systematic reporting of key process indicators and assessment and reporting of agreed clinically meaningful outcomes.

The existing heterogenous data prevent recommendation of any single approach to supportive care for patients with cancer. It is also impossible to limit specific components of care or to describe a definite effective model for the commissioning and delivery of supportive care to patients at scale. This heterogeneity may also account for some of the variation in service development and delivery that has been described, both in the UK and in France in recent years [6, 135]. There were however key features to supportive care services with the broadest benefits which were replicated across diverse settings and study designs included in this review. These included proactive delivery of coordinated multiprofessional care, routine screening of patients’ needs, and delivery of patient education including using digital technology. Hui, Hoge and Bruera (2021) described a conceptual framework for integrated supportive care services in which they described a model of routine access to a coordinated multidisciplinary team with systematic screening of needs and tailored access to specialists as a model which may provide the greatest improvements in clinical outcomes [136]. The findings of this review support this framework as we have been able to describe greatest plurality of benefits from models which do have a greater diversity of professionals available within a single coordinated service and embedded screening processes.

### The added value of supportive care

Despite the heterogeneity of the interventions and outcomes, the data from this review indicates that supportive care appears to play a role in facilitating individualised, patient-centred care. Whilst there is some evidence of cost-effectiveness within four included studies, the small number of studies and methodological heterogeneity suggest this is a promising area for further study. A major challenge for healthcare providers is no longer whether supportive care/ESC should be provided, but how. To deliver high-quality supportive care, services need to be adequately resourced [130]. Funding for supportive care is varied and inconsistent [6, 135]. A lack of resources (staffing or other) was highlighted as a barrier to implementation in this review. Within resource-constrained healthcare environments, business case planning and resource allocation is dependent on providing evidence of benefit for patients while also introducing positive impact for the wider economic system. Benefits for both patients and the healthcare system were evident in this review.

The most predominant outcome recorded in this scoping review was quality of life for patients with cancer receiving supportive care. Quality of life is an important component of patient experience, which sits alongside safety and effectiveness as a metric of quality of care [137]. For patients with cancer, quality of life is often as important as survival and disease progression and may even be associated with overall prognosis [138–140]. However, quality of life as an outcome may not be strong enough to result in commissioning of services, and as such was only mentioned twice in the 10-year health plan for England [141], perhaps limiting the appetite for the commissioning of models in this review which focus on improving quality of life alone.

Reducing unplanned and non-elective hospital admissions is a more dominant aim within the recent UK NHS 10-year health plan [141], with a view to improving patient flow and freeing up hospital beds for those patients who really need them. Hospital use and healthcare costs are outcomes described in this review with some evidence that certain models of supportive care can be cost-effective. Service models with the greatest economic impact described were those which are outpatient clinic and hospital-based. Significant cost savings were demonstrated through a reduction in unplanned admissions and length of stay in a study of multiprofessional enhanced supportive care clinics in cancer centres [8]. This in turn reflects findings within the existing literature of the strength of evidence supporting stand-alone clinics in reducing secondary care use in the palliative care setting alongside improvements in quality-of-life metrics, particularly when multiprofessional support is available within the outpatient clinic model [142, 143]. To meet the UK government’s ambition for achieving reduced hospital use from preventative community interventions, further research into the impact of models and components of care and interventions in the community setting is required.

The multiprofessional models of care included in this review, namely the supportive care/supportive oncology clinics and enhanced supportive care models, were those which provided the greatest reach of positive outcomes including improved quality of life [106] and psychological wellbeing [68], reduced symptom burden [7, 8, 57, 106], reduced hospital use, reduced cost [8, 49, 56], improved survival [47, 68], and reduced chemotherapy use towards the end of life [7].

### Integration between supportive care and oncology services

Key elements of integration were highlighted in this review, including systematic assessment, standardising referrals, patient and staff engagement, training and education and personalised care [3]. Further research, however, is needed regarding the most effective models of and elements supporting integration. For example, the effects of patient educational interventions were mixed, but increasing patient knowledge and awareness of services and interventions was highlighted as a facilitator to implementation. Screening for needs to support person-centred care was demonstrated, providing an opportunity for sustainable service delivery within healthcare systems with finite resources [144, 145]. This approach not only presents a pragmatic solution to enable provision of care to those most in need, whilst providing lower-intensity interventions for those with less complex needs, but is also supported by the findings of recent studies in palliative care. Temel et al. reported a stepped approach to the provision of palliative care based on screening at key transition points rather than providing routine access for all [144]. This study demonstrated non-inferiority in terms of quality of life for those undergoing the screening process but greater resource-efficiency in the delivery of the service. A further study by Zimmermann et al. implemented a screening process to facilitate access to early palliative care based on need, which demonstrated promising early results in being able to stratify palliative care resource [145]. Systematic screening, however, was not implemented across all studies included in this scoping review and questions remain about how to ensure equitable access to supportive care. Studies describing care navigation were more numerous [36, 55, 74, 98]. A blended approach of screening needs at scale combined with patient navigation may present an opportunity for an effective method of delivering sustainable community-based preventative care as outlined in the NHS-10-year plan [141], but this approach was not described in this review. Digital approaches to healthcare delivery have been a focus of much interest and innovation and are included in the models and outcomes in this review. Inequitable access and literacy were highlighted as barriers to digital approaches. The impact of digitally enabled supportive care on healthcare use was not evaluated and again presents an opportunity for research.

### Limitations

As the focus of scoping reviews is to provide evidence on the extent of the existing data, this scoping review demonstrates the breadth of interventions and outcomes measured and thus serves as a useful mapping of the data to inform future work refining interventions and services. With a focus on mapping all available data, a critical appraisal of the quality of the evidence was not carried out.

### Implications for ESC practice, policy, and research

The benefits across settings, diagnoses, and stages of illness highlighted by this review provide reassurance that there is merit in focusing on multi-professional supportive care/ESC to improve patient experience and outcomes across the entire patient disease journey. The published data present ESC as an effective model of care, with benefits to both patients (quality of life) and health systems (cost-savings, better integration).

To specify the key effective components of ESC, more evidence is needed from service users and their families, as well as from service providers, funders and commissioners. An update of Caulfield’s 2019 national evaluation of the models [6], scope, and reach of ESC services across the UK would provide insights into the iterative development and delivery of ESC and current scope, reach and funding. A better understanding of how to normalise supportive care/ESC as a key component of oncology care will underpin equitable integration within oncology and support accessible public health messaging, patient-centred decision-making and ultimately business case planning and service commissioning.

## Conclusion

The data from this review provides a wealth of evidence regarding the positive impact of supportive care models. The relative potency of the findings around multidisciplinary outpatient clinic models is clearly important in determining the future model of ESC services; however, there is also evidence for a wide variety of parallel interventions ranging from exercise to psycho-oncology. Digital models of care, education delivery, screening and patient navigation also showed promising outcomes, which may prove effective in facilitating future community-based care. Defining the models of care and delineating the key enablers to the delivery of quality, effective, efficient services will support the move towards adequately funded and resourced ESC with equitable access for all.

## Supplementary Information

Below is the link to the electronic supplementary material.

ESM 1 Supplementary Material 1 (DOCX 19.2 KB)

ESM 2 Supplementary Material 2 (DOCX 398 KB)

## Data Availability

No datasets were generated or analysed during the current study.
