# Supplementary material for: Models, outcomes, barriers, and facilitators of supportive care in cancer: a scoping review
Source: Support Care Cancer. 2026 Mar 27;34(4):376. doi: 10.1007/s00520-026-10613-1 (PMC13031207; doi:10.1007/s00520-026-10613-1)
Supplement: Supplementary file 1 — Supplementary Material 1 (DOCX 19.2 KB) [file 520_2026_10613_MOESM1_ESM.docx]

Supplementary Figure 1: **Scoping review search strategy, Inclusion and Exclusion criteria**

**Supportive care**

1. “Supportive care”
2. “Enhanced supportive care”
3. “Supportive oncology”
4. “service”
5. “multi-disciplinary team”
6. (1 OR 2 OR 3 OR 4 OR 5)

**Models**

1. model*
2. workforce
3. staff*
4. Integrat*
5. Design
6. (7 OR 8 OR 9 OR 10 OR 11)

**Outcomes**

1. impact*
2. benefit*
3. cost
4. result*
5. “objectives”
6. “outcomes”
7. (13 OR 14 OR 15 OR 16 OR 17 OR 18)

| **Inclusion** | **Exclusion** |
| --- | --- |
| Studies relating to cancer in adults | Studies relating to paediatric patients |
| Primary or secondary research design | Posters |
| Studies exploring service design or impact of interventions | Conference abstracts |
| Facilitators and barriers to establishment of services | Editorials (if no studies cited within which are in the inclusion criteria) |
| Quality of life if an outcome of studies | Studies relating to prevalence of needs rather than impact of service |
|  | Quality of life as a need, rather than outcome of intervention |
