# Supplementary material for: Models, outcomes, barriers, and facilitators of supportive care in cancer: a scoping review
Source: Support Care Cancer. 2026 Mar 27;34(4):376. doi: 10.1007/s00520-026-10613-1 (PMC13031207; doi:10.1007/s00520-026-10613-1)
Supplement: Supplementary file 2 — Supplementary Material 2 (DOCX 398 KB) [file 520_2026_10613_MOESM2_ESM.docx]

**Supplementary tables 1 and 2 and associated reference list**

**Supplementary Table 1: Characteristics of included studies**

| **First Author (Year of publication) Country** | **Models** | **Benefits, cost, outcomes** | **Facilitators, Barriers** | **Number of patients involved, n (intervention, control)** | **Cancer Diagnoses** | **Cancer stage** | **Study Methods** |
| --- | --- | --- | --- | --- | --- | --- | --- |
| **Randomised Controlled Trials** | | | | | | | |
| Aubin et al (2021) Canada[1] | X | X | X | 109 (54 intervention, 55 control) | Lung | non-surgical | Mixed methods |
| Badger et al (2020) USA[2] | X | X | X | 241, of which 230 survivor - caregiver dyads | Breast | Mixed | Quantitative |
| Baik S.H. et al (2024) USA[3] | X |  |  | 72 (36 intervention, 36 control) | Mixed | Unspecified | Quantitative |
| Bakitas et al (2015) USA[4] | X | X |  | 207 (104 intervention, 103 control) | all (mainly lung and UGI) | Incurable prog 6-24 months | Quantitative |
| Balci H. et al (2024) Turkey [5] | X | X |  | 81 (41 Intervention 40 control) | Breast | Unspecified | Quantitative |
| Bayati M et al (2019) Iran[6] | X | X | X | 64 (32 intervention) | Breast, prostate and colon | Stage I to III | Quantitative |
| Beikmoradi A et al (2015) Iran[7] | X | X |  | 85 (27 intervention, 30 control, 28 sham) | Mixed | Mixed | Quantitative |
| Belay W et al (2021) Ethiopia [8] | X | X |  | 114 (57 intervention, 57 control) | Breast | Mixed | Quantitative |
| Berglund et al (2006) Sweden [9] |  | X |  | 211 (160 intervention, 51 control) | Newly diagnosed Prostate | Unspecified | Quantitative |
| Bourke L et al (2013) UK[10] | X | X | X | 100 (50 intervention, 50 control) | Prostate | Receiving androgen depletion therapy | Quantitative |
| Chambers et al (2015) USA [11] | X | X |  | 189 (125 intervention, 64 control) | Prostate | Unspecified | Quantitative |
| Chambers S et al (2017) Australia[12] | X | X | X | 189 (94 intervention, 95 control) | Prostate | Metastatic and/or castrate resistant | Quantitative |
| Chung et al (2022) USA [13] | X |  |  | 42 (26 intervention, 16 control) | Pancreatic | Unspecified | Quantitative |
| Cormie P et al (2013) Australia [14] | X | X |  | 57 (29 intervention, 28 control) | Prostate | Unspecified (93% localised disease) | Quantitative |
| Cormie P et al (2013) Australia[15] | X | X | X | 20 (10 in intervention, 10 control) | Prostate | Stage IV | Quantitative |
| Dhawan S et al (2020) India [16] | X | X |  | 45 (22 intervention, 23 control) | Mixed (gynae, lung, head and neck) | Unspecified | Quantitative |
| Ebrahimabadi et al (2021) Iran [17] | X |  | X | 60 (30 intervention) | Mixed | Non-metastatic, undergoing chemotherapy | Quantitative |
| Eicher et al (2018) Switzerland [18] | X | X | X | 86 (41 intervention1, 45 intervention 2) | Solid tumour or lymphoma | Mixed, approximately 25% metatsatic | Mixed methods |
| Fatehi S et al (2019) Iran [19] | X | X |  | 118 (59 intervention, 59 control) | Breast | non- metastatic disease | Quantitative |
| Galvao D et al (2014) Australia and NZ [20] | X | X |  | 100 (50 intervention, 50 control) | Prostate >5 years after diagnosis | Survivors previously treated with androgen suppression and radiation | Quantitative |
| Geerse et al (2017) Netherlands [21] |  | X |  | 223 randomised | Lung | Mixed: newly diagnosed and recurrent | Quantitative |
| Ghanbari E et al (2021) Iran [22] | X | X |  | 82 (41 intervention, 41 control) | Breast | Non metastatic disease | Quantitative |
| Hiensch A. et al (2024) Europe / Australia [23] | X | X | X | 357 (178 intervention, 179 control) | Breast | Metastatic | Quantitative |
| Huang CC et al (2019) Taiwan [24] | X | X |  | 55 (27 intervention, 28 control) | Non small cell lung | stages IIIa, IIIb, or IV | Quantitative |
| Huri et al (2015) Turkey [25] | X |  |  | 34 (19 intervention, 15 control) | Prostate | Stage T3 or T4 with bone mets. | Quantitative |
| Jalambadani et al (2018) Iran [26] | X |  |  | 100 (50 intervention, 50 control) | Breast | Mixed | Quantitative |
| Jefford et al (2016) Australia [27] |  | X | X | 216 (106 intervention, 110 control) | Colorectal | Stage I to III | Quantitative |
| Ji et al (2019) South Korea [28] | X |  |  | 64 (32 intervention, 32 control) | Lung | Mixed | Quantitative |
| Jones et al (2013) UK[29] |  |  | X | 41 (21 intervention, 20 control) | Haematology and breast | Recurrent disease | Quantitative |
| Ke Y. and et al (2024) Singaopre [30] | X | X |  | 173 (83 intervention, 90 control) | Breast and gynaecological | Mixed | Quantitative |
| Koffi et al (2019) Ivory Coast [31] | X |  |  | 100 (51 intervention, 49 control) | Lymphoma | Mixed | Quantitative |
| Lu* et al (2021) China [32] | X | X |  | 328 (214 intervention, 114 control) | Oesophagogastric | Metastatic | Quantitative |
| Malmström et al (2016) Sweden [33] | X |  |  | 82 (41 intervention, 41 control) | Oesophagus | Post oesophagectomy or oesophagogastrectomy | Quantitative |
| Moghaddam et al (2016) Iran [34] | X |  | X | 60 (30 intervention and 30 control) | Breast | Stage I to III | Quantitative |
| Mohammed et al (2016) Egypt[35] | X |  |  | 60 | Breast | Unspecified | Quantitative |
| Nahm E.-S. et al (2025) USA [36] | X | X | X | 60 (31 intervention, 29 control) | Mixed | Mixed survivors | Quantitative |
| Noriega Es et al (2025) USA [37] | X |  | X | 288 (144 intervention, 144 control) | Breast, prostate and colorectal | Survivors stage 0 to III | Quantitative |
| Paterson et al (2018) scotland [38] | X | X | X | 48 (20 intervention, 28 control) | Prostate | Metastatic | Quantitative |
| Schenker et al (2021) USA [39] | X | X |  | 672 (336 intervention, 336 control) | Mixed: Solid tumours - lung, genito-urinary, brain, breast, gynaecological, gastrointestinal, hepatobiliary, head and neck, melanoma, sarcoma | Metastatic | Quantitative |
| Soto-Perez-de-Celis et al (2021) Mexico [40] | X |  |  | 134 (67 intervention, 67 control) | Solid tumour | Metastatic | Quantitative |
| Sun Z. and et al (2025) China [41] | X | X |  | 168 (84 intervention, 84 control) | Colorectal | Unspecified | Quantitative |
| Sussman et al (2018) Canada [42] | X |  |  | 193 (89 intervention, 103 control) | Breast and colorectal | Newly diagnosed, stage unspecified | Quantitative |
| Viamonte S et al (2024) Portugal [43] | X | X |  | 80 (40 intervention, 40 control) | Mixed | Survivors | Quantitative |
| Wolff J. a et al (2023) Germany [44] | X | X |  | 60 (38 intervention, 22 control) | Breast | Mixed | Quantitative |
| Yazicioglu et al (2024) Turkey [45] | X | X |  | 42 (21 intervention, 21 control) | Breast | Stage II or IIIB | Quantitative |
| **Non-randomised intervention studies with control arm** | | | | | | | |
| Aydin A. et al (2024) Turkey [46] | X | X |  | 90 (45 intervention 45 control) | Breast | Amenable to surgery | Quantitative |
| Beydoun N et al (2013) Australia [47] | X | X |  | 859 | Prostate | Receiving androgen suppression therapy | Quantitative |
| Daly et al (2013) USA [48] | X | X | X | 610 (278 intervention, 332 control) | Lung, gastrointestinal, or gynaecological | Stages III or IV | Quantitative |
| Elyasi F et al (2021) Iran [49] | X | X |  | 50 (15, 20 intervention arms, 15 control) | Breast | Non metastatic | Quantitative |
| Ha XTN et al (2019) Vietnam [50] | X | X |  | 115, (57 Intervention, 58 control) |  | Unspecified | Quantitative |
| NasiriZiba et al (2021) Iran [51] | X |  |  | 60 (30 each group) | Colorectal | Unspecified | Quantitative |
| Nasution et al (2020) Indonesia [52] | X |  |  | 108 (54 intervention, 54 control) | Gynecological | Stages I to V | Quantitative |
| **Prospective pre-post test single cohort intervention study** | | | | | | | |
| Fox et al (2019) Australia [53] |  | X | X | 18 approached, 13 received intervention and followed up. | Melanoma | Unspecified | Mixed methods |
| Gheyasi F et al (2019) Iran [54] | X | X |  | 50 | Acute Myeloid Leukaemia | acute or recurrent | Quantitative |
| Kristanti et al (2017) Indonesia [55] | X |  | X | 41 (30 completed study) | not specified | Stage III or IV | Quantitative |
| Sanchez S. et al (2024) Mexico [56] | X | X | X | 50 | Breast | Stages I-III | Mixed methods |
| Sun et al (2016) USA [57] | X |  |  | 11 | unknown | Stage III, IV | Quantitative |
| **Prospective single cohort study** | | | | | | | |
| Ben-Arye et al (2015) Israel [58] | X | X | X | 308 referred, (189 received intervention and follow up) | MIxed (mainly breast, gynae, GI) | MIxed receiving chemotherapy | Quantitative |
| Bergerot C et al (2025) Brazil and USA[59] | X |  | X | 50 | Renal cell | Metastatic | Quantitative |
| Chavarri-Guerra et al (2021) USA [60] | X |  | X | 45 | Mixed | Advanced | Quantitative |
| Cherifi et al (2022) France [61] | X | X | X | 102 | H&N | II, III, IV (67% stage IV) before starting chemoradiation | Quantitative |
| Coats V et al (2020) Canada [62] | X | X | X | 5 | Lung | Unresectable stage IIIb or IVb, receiving chemo | Quantitative |
| Cooksley et al (2018) UK [63] | X |  | X | 68 | Mixed | 73% curative intent | Quantitative |
| Dobos et al (2015) Germany [64] | X | X |  | 117 | Mixed , 65% breast | 10% metastatic | Quantitative |
| Hirayama H et al (2023) Japan [65] | X | X |  | 318 | Mixed | Mixed | Quantitative |
| Lafaro et al (2020) USA [66] | X |  | X | 45 patients and carer dyads | Mixed: gastrointestinal (colorectal, gastric, pancreas, liver) and lung | Unspecified | Quantitative |
| Le Boutill et al (2023) UK [67] | X |  |  | 51 | Myeloma, breast, prostate and lung | Treatable but not curable | Quantitative |
| Mercadante et al (2018) Italy [68] |  | X |  | 100 | Mixed | Unspecified | Quantitative |
| Rico et al (2017) Brazil [69] | X |  | X | 14 | Mixed : Hodgkin's lymphoma, cervical, breast, lung, ovarian, testicular, head and neck, tongue. | Unspecified | Qualitative |
| Scarborough et al (2018) USA [70] | X | X | X | 19 healthcare professionals | Not stated | Unspecified | Mixed methods |
| Taylor S. et al (2023) UK [71] |  | X | X | 184 patients, 67 caregivers | Mixed | Mixed | Mixed methods |
| Fox, Rina et al (2024) USA [72] | X |  |  | 7 | Mixed | Unspecified | Mixed methods |
| McMillan H et al (2025) USA [73] |  | X |  | 24 | Head and Neck | Survivors (disease free 2 years post DXT) | Quantitative |
| Shemesh B. et al (2025) Australia [74] | X |  |  | 331 | Prostate | Unspecified | Quantitative |
| Wood W.A. et al (2023) USA [75] | X | X |  | 50 | Mixed | Mixed | Quantitative |
| Monnery et al (2023) UK [76] |  | X |  | 4594 | Mixed: lung, melanoma, breast, head & neck, Upper GI, sarcoma | Unspecified | Quantitative |
| Kotronoulas et al (2018) UK [77] | X |  | X | 13 | Lung | Unspecified | Mixed methods |
| **Prospective cross-sectional study** | | | | | | | |
| Wong et al (2019) USA [78] | X |  | X | 200 | Gastrointestinal, Lung, Breast, Genitourinary, Gynecologic, Head and neck, Leukemia/lymphoma, Other | Locally advanced, recurrent, or metastatic. | Quantitative |
| Berezowska et al (2019) Netherlands [79] | X | X | X | 1091 patients offered intervention, 755 accepted, 120 questionnaire. 155 health care professionals invited to participate, 68 healthcare professionals participated | Breast, melanoma | Unspecified | Quantitative |
| Wall et al (2016) Australia [80] | X |  | X | 70 | Head and neck | Mixed | Quantitative |
| **Retrospective cohort study with comparison group** | | | | | | | |
| Benson S. et al (2023) UK [81] | X | X |  | 202 (101 intervention, 101 matched control) | Hepatopancreatobiliary | Incurable | Quantitative |
| Moffat G.T et al (2024) Canada [82] | X | X |  | 993 (482 pre-intervention, 511 post-intervention) | Pancreas | All | Quantitative |
| Monnery et al (2018) UK [83] | X |  | X | 50 | Mixed: Upper GI, hepatobiliary, skin, head and neck, brain | Unspecified | Quantitative |
| Rosenblum et al (2018) USA [84] | X | X |  | 200 (100 routine care vs 100 supportive oncology) | Mixed : head and neck, lung, GI, GU, gynaecological, breast | Advanced | Quantitative |
| Stewart E. et al (2023) UK [85] | X | X |  | 260 | Mixed | Mixed | Quantitative |
| Westfall et al (2018) United States [86] | X |  | X | 144 (48 intervention, 96 control) | Lung , head and neck . | Stage III or IV | Quantitative |
| Worster B. et al (2024) USA [87] | X | X |  | 422 (138 intervention, 86, 60 and 138 control groups) | Unknown | Unknown | Quantitative |
| **Retrospective single cohort study** | | | | | | | |
| Antonuzzo et al (2017) Italy [88] | X | X |  | 1358 in study cohort, 1275 in baseline cohort | Mixed | Mixed (58% metastatic) | Quantitative |
| Birkner D. et al (2024) Switzerland [89] | X |  | X | 363 | Mixed | Mixed | Quantitative |
| Blumenthal et al (2023) USA [90] | X |  |  | 114 | Mixed | Mixed | Quantitative |
| Jivraj et al (2023) Canada [91] |  |  |  | 319 | Gynae | Unspecified | Quantitative |
| Ke Y. and et al (2023) Singapore [92] | X |  | X | 1853 | Breast and gynaecological | Survivors | Quantitative |
| Meisenberg et al (2014) USA [93] | X | X |  | 340 visits from 330 patients | Mixed | Advanced disease, close to death. | Quantitative |
| Monnery et al (2022) UK [94] | X |  |  | 265 | Mixed | Metastatic | Quantitative |
| Price S.N. et al (2025) USA [95] | X |  | X | 315 | Lung | Survivors stage 0 to II | Quantitative |
| Sanchez-Da et al (2024) Mexico [96] | X |  |  | 100 | Mixed | Mixed | Quantitative |
| Walling et al (2016) United States [97] |  | X | X | 719 | Lung, colorectal, and pancreatic | Advanced stages (III and IV) | Quantitative |
| **Reviews** | | | | | | | |
| Calvo-Schimmel et al (2022) global [98] |  |  | X | 13 studies | Prostate | mixed | Mixed methods |
| White et al (2021) Australia, Netherlands, Scotland, US [99] |  |  | X | 14 to 668 patients | Mixed: prostate, colorectal, breast, melanoma, gynecological s. | Mixed | Mixed methods |
| Wijeratne et al (2021) US, Australia, Brazil, UK, France, Singapore [100] | X |  | X | 14 - 571 | Mixed | Mixed | Quantitative |
| **Surveys** | | | | | | | |
| Caulfield R et al (2024) UK [101] |  |  | X | 30 teams, 47 clinicians | NA | Unspecified | Mixed methods |
| Villareal-Garza et al (2020) Mexico [102] |  | X | X | 134 | Breast | Mixed | Mixed methods |
| Avancini A et al (2023) Italy [103] |  |  | X | 324 | Mixed | Mixed | Quantitative |
| **Qualitative studies** | | | | | | | |
| Afiyanti Y et al (2020) Indonesia [104] | X | X | X | 16 (and their partners) | Cervix | Stage II or III, completed treatment for 1 to 2 years | Qualitative |
| Avery et al (2021) Canada [105] |  |  | X | 57 patients, 11 caregivers | Mixed | Mixed | Qualitative |
| Bankole A. et al (2024) USA [106] | X |  | X | 8 clinicians | Acute Myeloid Leukaemia | Unspecified | Qualitative |
| Boltong et al (2016) UK, Australia, America [107] | X |  | X | 30 | Mixed (most prevalent breast) | Mixed | Qualitative |
| Burton et al (2013) UK [108] | X | X | X | 50 in 6 month period | Ovarian | Unspecified | Quantitative |
| Cormie P et al (2015) Australia [109] | X |  |  | 12 | Prostate | Unspecified | Qualitative |
| Evered et al (2022) USA [110] | X |  | X | 17 clinicians, 18 patients, 3 care partners |  | Unspecified | Qualitative |
| Montiel C. et al (2023) Canada [111] |  |  | X | 31 | Mixed | Mixed | Qualitative |
| Ralph et al (2020) Australia [112] |  |  | X | 21 | Prostate | Advanced (not explicitly stated) | Qualitative |
| Regnier et al (2017) France [113] |  |  | X | 36 | Breast | Unspecified | Qualitative |
| Young et al (2019) Scotland [114] |  | X |  | 20 | Breast, colorectal, cervical, thyroid, head and neck, lung, prostate, blood, ovarian | Unspecified | Mixed methods |
| **Mixed methods study** | | | | | | | |
| McLaughlin et al (2019) Canada [115] | X |  | X | 135 men and 72 partners | Prostate | Unspecified | Mixed methods |

**Supplementary Table 2 Models of care within included studies. The number of articles is included, [along with the references themselves within square brackets].**

**Note: when a study includes 2 or more professions working together, the study appears in all columns in which that profession is represented UNLESS the intervention is described as an MDT meeting or MDT clinic, which falls under “Multidisciplinary NOS”**

|  | **Palliative Care Doctor** | **Oncologist** | **Other Doctor** | **Palliative Care Nurse** | **Oncology Nurse** | **Other Nurse and Nurse Specialists** | **Physiotherapist** | **Occupational Therapist** | **Dietitian and/or nutritionist** | **Psychologist or psychotherapist** | **Exercise Physiologist and/or health coach** | **Care Navigator** | **Social Worker** | **Chaplain or other Spiritual support worker** | **Counsellor or Professional trained in mindfulness interventions** | **Multidisciplinary NOS** | **Cancer Survivors (peers)** | **Speech and Language Therapist** | **Pharmacist** |
| --- | --- | --- | --- | --- | --- | --- | --- | --- | --- | --- | --- | --- | --- | --- | --- | --- | --- | --- | --- |
| **Early Palliative Care** | 1 [4] |  |  |  |  | 1[4] |  |  |  |  |  |  |  |  |  |  |  |  |  |
| **Enhanced Supportive Care** | 2 [83,94] | 1 [32] |  |  |  | 2 [32,81] | 2[83,94] | 2[83,94] | 3[32,83,94] | 3 [32,83,94] |  |  | 2[83,94] |  |  |  |  |  |  |
| **Telephone Coaching** | 1[4] |  |  |  |  | 1[4] |  |  |  |  |  |  |  |  |  |  |  |  |  |
| **Supportive Oncology/Supportive Care Clinic** | 3[70,84,87] |  |  |  |  | 7[45,78,84,86,87,93,106] | 1[93] | 2[93,106] | 2[87,93] | 1[87] |  | 1[87] | 3 [84,87,93] |  |  |  |  |  | 2[86,87] |
| **Integrated Geriatric and Palliative Care Team** | 1[96] |  | 1[96] |  |  |  |  |  |  |  |  |  |  |  |  |  |  |  |  |
| **Inpatient Supportive Oncology Service** | 1[85] |  |  |  |  | 1[85] |  |  |  |  |  |  |  |  |  |  |  |  |  |
| **Pretherapeutic comprehensive assessment** |  | 1[61] |  |  | 1[61] |  |  |  | 1[61] |  |  |  |  |  |  |  |  |  |  |
| **Interprofessional Clinic** |  | 1[82] |  |  | 1[82] | 2[18,82] |  |  |  |  |  |  |  |  |  |  |  |  |  |
| **Integrative Oncology Programme** |  |  | 1[58] |  |  |  |  |  |  |  |  |  |  |  |  |  |  |  |  |
| **Virtual Supportive Care Clinic** |  |  | 1[110] |  |  |  |  |  | 1[110] |  |  |  | 1[110] |  |  |  |  |  |  |
| **Supportive or Palliative Care Clinic NOS** | 1[89] |  | 1[93] | 2[87,89] |  |  |  |  |  |  |  |  | 1[48] | 1[48] |  |  |  |  |  |
| **Cardiac Rehab** |  |  | 1[43] |  |  | 1[43] | 1[43] |  | 1[43] |  |  |  |  |  |  |  |  |  |  |
| **Systematic distress screening and tailored support for caregivers** |  |  | 1[1] |  | 1[1] |  |  |  |  |  |  |  |  |  |  |  |  |  |  |
| **Patient navigation intervention/ Care Coordination** |  |  |  |  | 1[79] | 2[31,42] |  |  | 1[60] | 2[40,60] |  | 4[31,37,40,60] |  |  |  |  |  |  |  |
| **Informational materials, holistic needs assessment, individualised self-management care plans, and group-based seminars** |  |  |  |  | 1[38] | 1[38] |  |  |  |  |  |  |  |  | 1[38] |  |  |  |  |
| **Couples based education intervention** |  |  |  |  | 1[41] |  |  |  |  |  |  |  |  |  |  |  |  |  |  |
| **Psychosexual Education** |  |  |  |  |  | 1[104] |  |  |  |  |  |  |  |  |  |  |  |  |  |
| **Digitally delivered education and support** |  |  |  |  |  | 5[22,24,46,56,110] |  |  |  |  |  |  |  |  |  |  |  |  |  |
| **Uncertainty Management Program** |  |  |  |  |  | 1[50] |  |  |  |  |  |  |  |  |  |  |  |  |  |
| **Care skills training for caregivers** |  |  |  |  |  | 1[55] |  |  |  |  |  |  |  |  |  |  |  |  |  |
| **Exercise Therapy** |  |  |  |  |  | 1[35] | 2[23,109] |  |  |  | 5[14,15,20,23,47] |  |  |  |  |  |  |  |  |
| **Spiritual Therapy** |  |  |  |  |  | 1[52] |  |  |  |  |  |  |  |  |  |  |  |  |  |
| **Cancer information service** |  |  |  |  |  | 1[107] |  |  |  |  |  |  |  |  |  |  |  |  |  |
| **Survivorship care, support and clinics** |  |  |  |  |  | 2[95,108] |  |  |  |  |  |  |  |  |  |  |  |  |  |
| **Telehealth and telephone support** |  |  |  |  |  | 2[11,17] |  |  | 1[33] |  |  |  | 1[2] |  | 1[11] |  | 1[11] |  |  |
| **Screening-based patient support services** |  |  |  |  |  | 3[30,77,92] |  |  |  | 1[92] |  |  | 1[92] |  |  |  |  |  | 1[92] |
| **Nurse-led face to face support and education** |  |  |  |  |  | 3[33,39,41] | 1[33] |  |  |  |  |  |  |  |  |  |  |  |  |
| **Prehab and Rehab** |  |  |  |  |  |  | 1[66] | 1[66] |  |  | 1[62] |  |  |  |  |  |  |  |  |
| **Cognitive behaviour therapy** |  |  |  |  |  |  |  | 1[25] |  |  |  |  |  |  | 1[12] |  |  |  |  |
| **Motivational interviewing/ health coaching** |  |  |  |  |  |  |  | 1[67] |  | 1[67] | 1[75] |  |  |  |  |  |  |  |  |
| **Combined speech, swallow and dietary intervention** |  |  |  |  |  |  |  |  | 1[80] |  |  |  |  |  |  |  |  | 1[80] |  |
| **Combined Dietary, exercise and lifestyle interventions** |  |  |  |  |  |  |  |  | 2[10,115] |  | 1[10] |  |  |  |  |  |  |  |  |
| **Day Therapy** |  |  |  |  |  |  |  |  |  | 1[64] |  |  |  |  | 1[64] |  |  |  |  |
| **Psychotherapy** |  |  |  |  |  |  |  |  |  | 1[8] |  |  |  |  |  |  |  |  |  |
| **Advisory MDT meeting** |  |  |  |  |  |  |  |  |  |  |  |  |  |  |  | 1[13] |  |  |  |

[1] Aubin M, Vézina L, Verreault R, Simard S, Desbiens J-F, Tremblay L, et al. A randomized clinical trial assessing a pragmatic intervention to improve supportive care for family caregivers of patients with lung cancer. Palliat Support Care 2021;19:146–53. https://doi.org/10.1017/S1478951520000711.

[2] Badger TA, Segrin C, Sikorskii A, Pasvogel A, Weihs K, Lopez AM, et al. Randomized controlled trial of supportive care interventions to manage psychological distress and symptoms in Latinas with breast cancer and their informal caregivers. Psychol Health 2020;35:87–106. https://doi.org/10.1080/08870446.2019.1626395.

[3] Baik SH, Clark K, Sanchez M, Loscalzo M, Celis A, Razavi M, et al. Usability and Preliminary Efficacy of an Adaptive Supportive Care System for Patients With Cancer: Pilot Randomized Controlled Trial. JMIR Cancer 2024;10:e49703. https://doi.org/10.2196/49703.

[4] Bakitas MA, Tosteson TD, Li Z, Lyons KD, Hull JG, Li Z, et al. Early Versus Delayed Initiation of Concurrent Palliative Oncology Care: Patient Outcomes in the ENABLE III Randomized Controlled Trial. Journal of Clinical Oncology 2015;33:1438–45. https://doi.org/10.1200/JCO.2014.58.6362.

[5] BALCI H, FAYDALI S. The Effect of Education Performed Using Mobile Application on Supportive Care Needs and Quality of Life in Women with Breast Cancer: Randomized Controlled Trial. Semin Oncol Nurs 2024;40:151684. https://doi.org/10.1016/j.soncn.2024.151684.

[6] Bayati M, Molavynejad S, Taheri N, Cheraghian B. Investigating the effect of Integrated Educational Program on the Quality of Life among Cancer Patients: A Clinical Trial Study. Asian Pacific Journal of Cancer Prevention 2019;20:3457–63. https://doi.org/10.31557/APJCP.2019.20.11.3457.

[7] Beikmoradi A, Najafi F, Roshanaei GA, Pour Esmaeil Z, Khatibian M, Ahmadi A. Acupressure and Anxiety in Cancer Patients. Iran Red Crescent Med J 2015;17. https://doi.org/10.5812/ircmj.25919.

[8] Belay W, kaba M, Labisso WL, Tigeneh W, Sahile Z, Zergaw A, et al. The effect of interpersonal psychotherapy on quality of life among breast cancer patients with common mental health disorder: a randomized control trial at Tikur Anbessa Specialized Hospital. Supportive Care in Cancer 2022;30:965–72. https://doi.org/10.1007/s00520-021-06508-y.

[9] Berglund G, Petersson L-M, Eriksson KC, Wallenius I, Roshanai A, Nordin KM, et al. “Between Men”: A psychosocial rehabilitation programme for men with prostate cancer. Acta Oncol (Madr) 2007;46:83–9. https://doi.org/10.1080/02841860600857326.

[10] Bourke L, Gilbert S, Hooper R, Steed LA, Joshi M, Catto JWF, et al. Lifestyle Changes for Improving Disease-specific Quality of Life in Sedentary Men on Long-term Androgen-Deprivation Therapy for Advanced Prostate Cancer: A Randomised Controlled Trial. Eur Urol 2014;65:865–72. https://doi.org/10.1016/j.eururo.2013.09.040.

[11] Chambers SK, Occhipinti S, Schover L, Nielsen L, Zajdlewicz L, Clutton S, et al. A randomised controlled trial of a couples‐based sexuality intervention for men with localised prostate cancer and their female partners. Psychooncology 2015;24:748–56. https://doi.org/10.1002/pon.3726.

[12] Chambers SK, Occhipinti S, Foley E, Clutton S, Legg M, Berry M, et al. Mindfulness-Based Cognitive Therapy in Advanced Prostate Cancer: A Randomized Controlled Trial. Journal of Clinical Oncology 2017;35:291–7. https://doi.org/10.1200/JCO.2016.68.8788.

[13] Chung V, Sun V, Ruel N, Smith TJ, Ferrell BR. Improving Palliative Care and Quality of Life in Pancreatic Cancer Patients. J Palliat Med 2022;25:720–7. https://doi.org/10.1089/jpm.2021.0187.

[14] Cormie P, Newton RU, Taaffe DR, Spry N, Joseph D, Akhlil Hamid M, et al. Exercise maintains sexual activity in men undergoing androgen suppression for prostate cancer: a randomized controlled trial. Prostate Cancer Prostatic Dis 2013;16:170–5. https://doi.org/10.1038/pcan.2012.52.

[15] Cormie P, Newton RU, Spry N, Joseph D, Taaffe DR, Galvão DA. Safety and efficacy of resistance exercise in prostate cancer patients with bone metastases. Prostate Cancer Prostatic Dis 2013;16:328–35. https://doi.org/10.1038/pcan.2013.22.

[16] Dhawan S, Andrews R, Kumar L, Wadhwa S, Shukla G. A Randomized Controlled Trial to Assess the Effectiveness of Muscle Strengthening and Balancing Exercises on Chemotherapy-Induced Peripheral Neuropathic Pain and Quality of Life Among Cancer Patients. Cancer Nurs 2020;43:269–80. https://doi.org/10.1097/NCC.0000000000000693.

[17] Ebrahimabadi M, Rafiei F, Nejat N. Can tele-nursing affect the supportive care needs of patients with cancer undergoing chemotherapy? A randomized controlled trial follow-up study. Supportive Care in Cancer 2021;29:5865–72. https://doi.org/10.1007/s00520-021-06056-5.

[18] Eicher M, Ribi K, Senn‐Dubey C, Senn S, Ballabeni P, Betticher D. Interprofessional, psycho‐social intervention to facilitate resilience and reduce supportive care needs for patients with cancer: Results of a noncomparative, randomized phase II trial. Psychooncology 2018;27:1833–9. https://doi.org/10.1002/pon.4734.

[19] Fatehi S, Maasoumi R, Atashsokhan G, Hamidzadeh A, Janbabaei G, Mirrezaie SM. The effects of psychosexual counseling on sexual quality of life and function in Iranian breast cancer survivors: a randomized controlled trial. Breast Cancer Res Treat 2019;175:171–9. https://doi.org/10.1007/s10549-019-05140-z.

[20] Galvão DA, Spry N, Denham J, Taaffe DR, Cormie P, Joseph D, et al. A Multicentre Year-long Randomised Controlled Trial of Exercise Training Targeting Physical Functioning in Men with Prostate Cancer Previously Treated with Androgen Suppression and Radiation from TROG 03.04 RADAR. Eur Urol 2014;65:856–64. https://doi.org/10.1016/j.eururo.2013.09.041.

[21] Geerse OP, Hoekstra-Weebers JEHM, Stokroos MH, Burgerhof JGM, Groen HJM, Kerstjens HAM, et al. Structural distress screening and supportive care for patients with lung cancer on systemic therapy: A randomised controlled trial. Eur J Cancer 2017;72:37–45. https://doi.org/10.1016/j.ejca.2016.11.006.

[22] Ghanbari E, Yektatalab S, Mehrabi M. Effects of Psychoeducational Interventions Using Mobile Apps and Mobile-Based Online Group Discussions on Anxiety and Self-Esteem in Women With Breast Cancer: Randomized Controlled Trial. JMIR Mhealth Uhealth 2021;9:e19262. https://doi.org/10.2196/19262.

[23] Hiensch AE, Depenbusch J, Schmidt ME, Monninkhof EM, Pelaez M, Clauss D, et al. Supervised, structured and individualized exercise in metastatic breast cancer: a randomized controlled trial. Nat Med 2024;30:2957–66. https://doi.org/10.1038/s41591-024-03143-y.

[24] Huang C-C, Kuo H-P, Lin Y-E, Chen S-C. Effects of a Web-based Health Education Program on Quality of Life and Symptom Distress of Initially Diagnosed Advanced Non-Small Cell Lung Cancer Patients: A Randomized Controlled Trial. Journal of Cancer Education 2019;34:41–9. https://doi.org/10.1007/s13187-017-1263-y.

[25] Huri M, Huri E, Kayihan H, Altuntas O. Effects of occupational therapy on quality of life of patients with metastatic prostate cancer. Saudi Med J 2015;36:954–61. https://doi.org/10.15537/smj.2015.8.11461.

[26] Jalambadani Z, Borji A. Effectiveness of Mindfulness-Based Art Therapy on Healthy Quality of Life in Women with Breast Cancer. Asia Pac J Oncol Nurs 2019;6:193–7. https://doi.org/10.4103/apjon.apjon_36_18.

[27] Jefford M, Gough K, Drosdowsky A, Russell L, Aranda S, Butow P, et al. A Randomized Controlled Trial of a Nurse-Led Supportive Care Package (SurvivorCare) for Survivors of Colorectal Cancer. Oncologist 2016;21:1014–23. https://doi.org/10.1634/theoncologist.2015-0533.

[28] Ji W, Kwon H, Lee S, Kim S, Hong JS, Park YR, et al. Mobile Health Management Platform–Based Pulmonary Rehabilitation for Patients With Non–Small Cell Lung Cancer: Prospective Clinical Trial. JMIR Mhealth Uhealth 2019;7:e12645. https://doi.org/10.2196/12645.

[29] Jones L, FitzGerald G, Leurent B, Round J, Eades J, Davis S, et al. Rehabilitation in Advanced, Progressive, Recurrent Cancer: A Randomized Controlled Trial. J Pain Symptom Manage 2013;46:315-325.e3. https://doi.org/10.1016/j.jpainsymman.2012.08.017.

[30] Ke Y, Neo PSH, Yang GM, Neo SH-S, Tan YY, Tan YP, et al. Impact of a Multidisciplinary Supportive Care Model Using Distress Screening at an Asian Ambulatory Cancer Center: A Cluster Randomized Controlled Trial. JCO Oncol Pract 2024;20:1207–18. https://doi.org/10.1200/OP.23.00505.

[31] Koffi KG, Silué DA, Laurent C, Boidy K, Koui S, Compaci G, et al. AMAFRICA, a patient-navigator program for accompanying lymphoma patients during chemotherapy in Ivory Coast: a prospective randomized study. BMC Cancer 2019;19:1247. https://doi.org/10.1186/s12885-019-6478-3.

[32] Lu Z, Fang Y, Liu C, Zhang X, Xin X, He Y, et al. Early Interdisciplinary Supportive Care in Patients With Previously Untreated Metastatic Esophagogastric Cancer: A Phase III Randomized Controlled Trial. Journal of Clinical Oncology 2021;39:748–56. https://doi.org/10.1200/JCO.20.01254.

[33] Malmström M, Ivarsson B, Klefsgård R, Persson K, Jakobsson U, Johansson J. The effect of a nurse led telephone supportive care programme on patients’ quality of life, received information and health care contacts after oesophageal cancer surgery—A six month RCT-follow-up study. Int J Nurs Stud 2016;64:86–95. https://doi.org/10.1016/j.ijnurstu.2016.09.009.

[34] Moghaddam Tabrizi F, Alizadeh S. Family Intervention Based on the FOCUS Program Effects on Cancer Coping in Iranian Breast Cancer Patients: a Randomized Control Trial. Asian Pac J Cancer Prev 2018;19:1523–8. https://doi.org/10.22034/APJCP.2018.19.6.1523.

[35] Mohammed S. Effects of Exercise Intervention on Pain, Shoulder Movement, and  Functional Status in Women after Breast Cancer Surgery: A  Randomized Controlled Trial. Journal of Education and Practice 2016;7.

[36] Nahm E-S, McQuaige M, Steacy K, Zhu S, Seong H. The Impact of a Digital Cancer Survivorship Patient Engagement Toolkit on Older Cancer Survivors’ Health Outcomes. CIN: Computers, Informatics, Nursing 2025;43. https://doi.org/10.1097/CIN.0000000000001199.

[37] Noriega Esquives BS, Moreno PI, Munoz E, Lad TE, Hollowell CMP, Benzo RM, et al. Effects of a culturally tailored patient navigation program on unmet supportive care needs in Hispanic/Latino cancer survivors: A randomized controlled trial. Cancer 2025;131. https://doi.org/10.1002/cncr.35626.

[38] Paterson C, Primeau C, Nabi G. A pilot randomised controlled trial of a multimodal supportive care (ThriverCare) intervention for managing unmet supportive care needs in men with metastatic prostate cancer on hormonal treatment and their partner/caregivers. European Journal of Oncology Nursing 2018;37:65–73. https://doi.org/10.1016/j.ejon.2018.10.007.

[39] Schenker Y, Althouse AD, Rosenzweig M, White DB, Chu E, Smith KJ, et al. Effect of an Oncology Nurse–Led Primary Palliative Care Intervention on Patients With Advanced Cancer. JAMA Intern Med 2021;181:1451. https://doi.org/10.1001/jamainternmed.2021.5185.

[40] Soto-Perez-de-Celis E, Chavarri-Guerra Y, Ramos-Lopez WA, Alcalde-Castro J, Covarrubias-Gomez A, Navarro-Lara Á, et al. Patient Navigation to Improve Early Access to Supportive Care for Patients with Advanced Cancer in Resource-Limited Settings: A Randomized Controlled Trial. Oncologist 2021;26:157–64. https://doi.org/10.1002/onco.13599.

[41] Sun Z, Zhang Y, Yang X, Wang Y, Li Q, Zhao J. A randomized controlled trial of an intervention for unmet supportive care needs addressing colorectal cancer couples. European Journal of Oncology Nursing 2025;74:102805. https://doi.org/10.1016/j.ejon.2025.102805.

[42] Sussman J, Bainbridge D, Whelan TJ, Brazil K, Parpia S, Wiernikowski J, et al. Evaluation of a specialized oncology nursing supportive care intervention in newly diagnosed breast and colorectal cancer patients following surgery: a cluster randomized trial. Supportive Care in Cancer 2017. https://doi.org/10.1007/s00520-017-3981-4.

[43] Viamonte SG, Tavares A, Alves AJ, Joaquim A, Vilela E, Capela A, et al. Cost-effectiveness analysis of a cardio-oncology rehabilitation framework compared to an exercise intervention for cancer survivors with high cardiovascular risk. Eur J Prev Cardiol 2024. https://doi.org/10.1093/eurjpc/zwae181.

[44] Wolff J, Wuelfing P, König A, Ehrl B, Damsch J, Smollich M, et al. App-Based Lifestyle Coaching (PINK!) Accompanying Breast Cancer Patients and Survivors to Reduce Psychological Distress and Fatigue and Improve Physical Activity: A Feasibility Pilot Study. Breast Care 2023;18:354–65. https://doi.org/10.1159/000531495.

[45] Yazicioglu Kucuk B, Zorba Bahceli P. The Effects of Nurse-Led Supportive Care Program on Quality of Life in Women with Breast Cancer Receiving Adjuvant Chemotherapy: A Randomized Controlled Pilot Study. Semin Oncol Nurs 2024;40:151609. https://doi.org/10.1016/j.soncn.2024.151609.

[46] Aydin A, Gürsoy A. Nurse-Led Mobile App Effect on Quality of Life in Breast Cancer Patients After Surgery: Nonrandomized Controlled Prospective Cohort Study (Step 3). Cancer Nurs 2024. https://doi.org/10.1097/NCC.0000000000001418.

[47] Beydoun N, Bucci JA, Chin YS, Spry N, Newton R, Galvão DA. Prospective study of exercise intervention in prostate cancer patients on androgen deprivation therapy. J Med Imaging Radiat Oncol 2014;58:369–76. https://doi.org/10.1111/1754-9485.12115.

[48] Daly BJ, Douglas SL, Gunzler D, Lipson AR. Clinical Trial of a Supportive Care Team for Patients With Advanced Cancer. J Pain Symptom Manage 2013;46:775–84. https://doi.org/10.1016/j.jpainsymman.2012.12.008.

[49] Elyasi F FTFZMMMCSBM. Cognitive-Behavioral Therapy and Hypnosis Intervention on Anxiety, Depression, and Quality of Life in Patients with Breast Cancer Undergoing Chemotherapy: A Clinical Trial. Middle East J Cancer 2021;12.

[50] Ha XTN, Thanasilp S, Thato R. The Effect of Uncertainty Management Program on Quality of Life Among Vietnamese Women at 3 Weeks Postmastectomy. Cancer Nurs 2019;42:261–70. https://doi.org/10.1097/NCC.0000000000000597.

[51] NasiriZiba F, Kanani S. The Effect of Education with a Family-Centered and Client-Centered Approach on the Quality of Life in Patients with Stoma. J Caring Sci 2019;9:225–30. https://doi.org/10.34172/jcs.2020.034.

[52] Nasution LA, Afiyanti Y, Kurniawati W. Effectiveness of Spiritual Intervention toward Coping and Spiritual Well-being on Patients with Gynecological Cancer. Asia Pac J Oncol Nurs 2020;7:273–9. https://doi.org/10.4103/apjon.apjon_4_20.

[53] Fox J, Janda M, Bennett F, Langbecker D. An outreach telephone program for advanced melanoma supportive care: Acceptability and feasibility. European Journal of Oncology Nursing 2019;42:110–5. https://doi.org/10.1016/j.ejon.2019.08.010.

[54] Gheyasi F, Baraz S, Malehi A, Ahmadzadeh A, Salehi R, Vaismoradi M. Effect of the Walking Exercise Program on Cancer-Related Fatigue in Patients with Acute Myeloid Leukemia Undergoing Chemotherapy. Asian Pacific Journal of Cancer Prevention 2019;20:1661–6. https://doi.org/10.31557/APJCP.2019.20.6.1661.

[55] Kristanti MS, Setiyarini S, Effendy C. Enhancing the quality of life for palliative care cancer patients in Indonesia through family caregivers: a pilot study of basic skills training. BMC Palliat Care 2017;16:4. https://doi.org/10.1186/s12904-016-0178-4.

[56] Contreras Sánchez SE, Doubova S V, Martinez Vega IP, Grajales Álvarez R, Villalobos Valencia R, Dip Borunda AK, et al. Addressing the unmet needs of women with breast cancer in Mexico: a non-randomised pilot study of the digital ePRO intervention. BMJ Open 2024;14:e087240. https://doi.org/10.1136/bmjopen-2024-087240.

[57] Sun V, Ruel N, Chung V, Singh G, Leong L, Fakih M, et al. Pilot study of an interdisciplinary supportive care planning intervention in pancreatic cancer. Supportive Care in Cancer 2016;24:3417–24. https://doi.org/10.1007/s00520-016-3155-9.

[58] Ben-Arye E, Aharonson ML, Schiff E, Samuels N. Alleviating gastro-intestinal symptoms and concerns by integrating patient-tailored complementary medicine in supportive cancer care. Clinical Nutrition 2015;34:1215–23. https://doi.org/10.1016/j.clnu.2014.12.011.

[59] Bergerot CD, Bergerot PG, Philip EJ, Malhotra J, Castro D V, Govindarajan A, et al. Feasibility and acceptability of a mindfulness app-based intervention among patients with metastatic renal cell carcinoma: a multinational study. Oncologist 2025;30. https://doi.org/10.1093/oncolo/oyae309.

[60] Chávarri-Guerra Y, Ramos-López WA, Covarrubias-Gómez A, Sánchez-Román S, Quiroz-Friedman P, Alcocer-Castillejos N, et al. Providing Supportive and Palliative Care Using Telemedicine for Patients with Advanced Cancer During the COVID-19 Pandemic in Mexico. Oncologist 2021;26:e512–5. https://doi.org/10.1002/onco.13568.

[61] Cherifi F, Villemin M, Bisiaux F, Johnson A, Solem Laviec H, Rambeau A. Impact of Early Supportive Care Assessment on treatment decision in head and neck cancer before concomitant chemoradiotherapy. Supportive Care in Cancer 2022;30:6545–53. https://doi.org/10.1007/s00520-022-07078-3.

[62] Coats V, Moffet H, Vincent C, Simard S, Tremblay L, Maltais F, et al. Feasibility of an eight-week telerehabilitation intervention for patients with unresectable thoracic neoplasia receiving chemotherapy: A pilot study. Canadian Journal of Respiratory, Critical Care, and Sleep Medicine 2020;4:14–24. https://doi.org/10.1080/24745332.2019.1575703.

[63] Cooksley T, Campbell G, Al-Sayed T, LaMola L, Berman R. A novel approach to improving ambulatory outpatient management of low risk febrile neutropenia: an Enhanced Supportive Care (ESC) clinic. Supportive Care in Cancer 2018;26:2937–40. https://doi.org/10.1007/s00520-018-4194-1.

[64] Dobos G, Overhamm T, Büssing A, Ostermann T, Langhorst J, Kümmel S, et al. Integrating mindfulness in supportive cancer care: a cohort study on a mindfulness-based day care clinic for cancer survivors. Supportive Care in Cancer 2015;23:2945–55. https://doi.org/10.1007/s00520-015-2660-6.

[65] Hirayama H, Satomi E, Kizawa Y, Miyazaki M, Tagami K, Sekine R, et al. The effect of palliative care team intervention and symptom improvement using patient-reported outcomes: a multicenter prospective observational study. Supportive Care in Cancer 2023;31:439. https://doi.org/10.1007/s00520-023-07912-2.

[66] Lafaro KJ, Raz DJ, Kim JY, Hite S, Ruel N, Varatkar G, et al. Pilot study of a telehealth perioperative physical activity intervention for older adults with cancer and their caregivers. Supportive Care in Cancer 2020;28:3867–76. https://doi.org/10.1007/s00520-019-05230-0.

[67] Le Boutillier C, Jeyasingh-Jacob J, Jones L, King A, Archer S, Urch C. Improving personalised care and support planning for people living with treatable-but-not-curable cancer. BMJ Open Qual 2023;12:e002322. https://doi.org/10.1136/bmjoq-2023-002322.

[68] Mercadante S, Marchetti P, Adile C, Caruselli A, Ferrera P, Costanzi A, et al. Characteristics and care pathways of advanced cancer patients in a palliative-supportive care unit and an oncological ward. Supportive Care in Cancer 2018;26:1961–6. https://doi.org/10.1007/s00520-017-4037-5.

[69] Rico TM, dos Santos Machado K, Fernandes VP, Madruga SW, Noguez PT, Barcelos CRG, et al. Text Messaging (SMS) Helping Cancer Care in Patients Undergoing Chemotherapy Treatment: a Pilot Study. J Med Syst 2017;41:181. https://doi.org/10.1007/s10916-017-0831-3.

[70] Scarborough B, Goldhirsch S, Chai E. Building a Supportive Oncology Practice that Impacts Emergency Department Visits, Hospice Utilization, and Hospital Admission. J Palliat Med 2018;21:1499–503. https://doi.org/10.1089/jpm.2017.0709.

[71] Taylor S, Vercell A, Sawyer C, Khatoon B, Coomber-Moore J, Yorke J, et al. Enhanced supportive care: prospective cohort study of oncology patients and caregivers. BMJ Support Palliat Care 2023. https://doi.org/10.1136/spcare-2023-004231.

[72] Fox RS, Torres TK, Badger TA, Katsanis E, Yang D, Sanford SD, et al. Delivering a Group-Based Quality of Life Intervention to Young Adult Cancer Survivors via a Web Platform: Feasibility Trial. JMIR Cancer 2024;10:e58014–e58014. https://doi.org/10.2196/58014.

[73] McMillan H, Warneke CL, Buoy S, Porsche C, Savage K, Lai SY, et al. Manual Therapy for Fibrosis-Related Late Effect Dysphagia in Head and Neck Cancer Survivors. JAMA Otolaryngology–Head & Neck Surgery 2025;151:319. https://doi.org/10.1001/jamaoto.2024.5157.

[74] Shemesh B, Opie JL, Dunn RL, Mclaughlin G, Argawal V, Pomery A, et al. Developing, Piloting and Evaluating a Patient Support Portal for Men With Prostate Cancer in Victoria: An Action Research Study. Health Expectations 2025;28. https://doi.org/10.1111/hex.70149.

[75] Wood WA, Bailey C, Castrogivanni B, Mehedint D, Bryant AL, Lavin K, et al. Piloting <scp>HealthScore</scp> : Feasibility and acceptability of a clinically integrated health coaching program for people living with cancer. Cancer Med 2023;12:8804–14. https://doi.org/10.1002/cam4.5625.

[76] Monnery D, Tredgett K, Hooper D, Barringer G, Munton A, Thomas M, et al. Delivery Models and Health Economics of Supportive Care Services in England: A Multicentre Analysis. Clin Oncol 2023;35:e395–403. https://doi.org/10.1016/j.clon.2023.03.002.

[77] Kotronoulas G, Papadopoulou C, Simpson MF, McPhelim J, Mack L, Maguire R. Using patient-reported outcome measures to deliver enhanced supportive care to people with lung cancer: feasibility and acceptability of a nurse-led consultation model. Supportive Care in Cancer 2018;26:3729–37. https://doi.org/10.1007/s00520-018-4234-x.

[78] Wong A, Vidal M, Prado B, Hui D, Epner M, Balankari VR, et al. Patients’ Perspective of Timeliness and Usefulness of an Outpatient Supportive Care Referral at a Comprehensive Cancer Center. J Pain Symptom Manage 2019;58:275–81. https://doi.org/10.1016/j.jpainsymman.2019.04.027.

[79] Berezowska A, Passchier E, Bleiker E. Evaluating a professional patient navigation intervention in a supportive care setting. Supportive Care in Cancer 2019;27:3281–90. https://doi.org/10.1007/s00520-018-4622-2.

[80] Wall LR, Cartmill B, Ward EC, Hill AJ, Isenring E, Porceddu S V. Evaluation of a weekly speech pathology/dietetic service model for providing supportive care intervention to head and neck cancer patients and their carers during (chemo)radiotherapy. Supportive Care in Cancer 2016;24:1227–34. https://doi.org/10.1007/s00520-015-2912-5.

[81] Benson S, Wong H, Olsson-Brown A, Coyle S, Monnery D. Palliative care clinical nurse specialists leading enhanced supportive care in hepatopancreatobiliary cancer. Int J Palliat Nurs 2023;29:129–36. https://doi.org/10.12968/ijpn.2023.29.3.129.

[82] Moffat GT, Coyne Z, Albaba H, Aung KL, Dodd A, Espin-Garcia O, et al. Impact of an Inter-Professional Clinic on Pancreatic Cancer Outcomes: A Retrospective Cohort Study. Current Oncology 2024;31:2589–97. https://doi.org/10.3390/curroncol31050194.

[83] Monnery D, Benson S, Griffiths A, Cadwallader C, Hampton-Matthews J, Coackley A, et al. Multi-professional-delivered enhanced supportive care improves quality of life for patients with incurable cancer. Int J Palliat Nurs 2018;24:510–4. https://doi.org/10.12968/ijpn.2018.24.10.510.

[84] Rosenblum R, Huo R, Scarborough B, Goldstein N, Smith CB. Comparison of Quality Oncology Practice Initiative Metrics in Solid Tumor Oncology Clinic With or Without Concomitant Supportive Oncology Consultation. J Oncol Pract 2018;14:e786–93. https://doi.org/10.1200/JOP.18.00380.

[85] Stewart E, Tavabie S, McGovern C, Round A, Shaw L, BAss S, et al. Cancer centre supportive oncology service: health economic evaluation. BMJ Support Palliat Care 2023;13:228–33. https://doi.org/10.1136/spcare-2022-003716.

[86] Westfall K, Moore D, Meeneghan M, Jarr S, Valgus J, Bernard S. The Impact on Resource Utilization of Supportive Care Consults on Patients at the University of North Carolina Hospital, 2010–2012. J Palliat Med 2018;21:216–9. https://doi.org/10.1089/jpm.2016.0482.

[87] Worster B, Zhu Y, Garber G, Kieffer S, Smith‐McLallen A. The impact of outpatient supportive oncology on cancer care cost and utilization. Cancer 2024;130:2848–55. https://doi.org/10.1002/cncr.35332.

[88] Antonuzzo A, Vasile E, Sbrana A, Lucchesi M, Galli L, Brunetti IM, et al. Impact of a supportive care service for cancer outpatients: management and reduction of hospitalizations. Preliminary results of an integrated model of care. Supportive Care in Cancer 2017;25:209–12. https://doi.org/10.1007/s00520-016-3403-z.

[89] Birkner DR, Schettle M, Feuz M, Blum D, Hertler C. Outpatient Palliative Care Service Involvement: A Five-Year Experience from a Tertiary Hospital in Switzerland. Palliat Med Rep 2024;5:10–9. https://doi.org/10.1089/pmr.2023.0052.

[90] Blumenthaler AN, Bruera E, Badgwell BD. Palliative and Supportive Care Consultation for Patients With Malignant Gastrointestinal Obstruction is Associated With Broad Interdisciplinary Management. Ann Surg 2023;277:284–90. https://doi.org/10.1097/SLA.0000000000004974.

[91] Jivraj N, Lee YC, Tinker L, Bowering V, Ferguson SE, Croke J, et al. Management of Malignant Bowel Obstruction. J Nurs Care Qual 2023;38:69–75. https://doi.org/10.1097/NCQ.0000000000000661.

[92] Ke Y, Tan YY, Neo PSH, Yang GM, Loh KW-J, Ho S, et al. &lt;p class="MsoNormal"&gt;&lt;span lang="EN-GB"&gt;Implementing an Inclusive, Multidisciplinary Supportive Care Model to Provide Integrated Care to Breast and Gynaecological Cancer Survivors: A Case Study at an Asian Ambulatory Cancer Centre&lt;/span&gt;&lt;/p&gt; Int J Integr Care 2023;23:14. https://doi.org/10.5334/ijic.6480.

[93] Meisenberg B, Graze L, Brady-Copertino C. A supportive care clinic for cancer patients embedded within an oncology practice. J Community Support Oncol 2014;12:205–8. https://doi.org/10.12788/jcso.0049.

[94] Monnery D, Liu Y, Griffiths A, Lockhart J, Coyle S, Olsson-Brown A. Multidisciplinary supportive care in cancer: cost analysis. BMJ Support Palliat Care 2024;14:e512–5. https://doi.org/10.1136/spcare-2022-004135.

[95] Price SN, Willis AR, Hensley A, Hyson J, Sohl SJ, D’Agostino RB, et al. Implementation and Retrospective Examination of a Lung Cancer Survivorship Clinic in a Comprehensive Cancer Center. Clin Lung Cancer 2025;26:e41–54. https://doi.org/10.1016/j.cllc.2024.09.008.

[96] Sánchez-Dávila JN, Verástegui EL, Peña-Nieves A, Allende-Pérez SR. Integration of the geriatric palliative care in oncological care of elderly patient with cancer. Palliat Support Care 2024;22:792–800. https://doi.org/10.1017/S1478951524000294.

[97] Walling AM, Tisnado D, Ettner SL, Asch SM, Dy SM, Pantoja P, et al. Palliative Care Specialist Consultation Is Associated With Supportive Care Quality in Advanced Cancer. J Pain Symptom Manage 2016;52:507–14. https://doi.org/10.1016/j.jpainsymman.2016.04.005.

[98] Calvo-Schimmel A, Newman SD, Sterba KR, Miaskowski C, Qanungo S. Barriers and Facilitators to Supportive Care Implementation in Advanced Disease Prostate Cancer Survivors. Cancer Nurs 2022;45:E782–800. https://doi.org/10.1097/NCC.0000000000001051.

[99] White VM, Pejoski N, Vella E, Skaczkowski G, Ugalde A, Yuen EYN, et al. Improving access to cancer information and supportive care services: A systematic review of mechanisms applied to link people with cancer to psychosocial supportive care services. Psychooncology 2021;30:1603–25. https://doi.org/10.1002/pon.5744.

[100] Wijeratne DT, Bowman M, Sharpe I, Srivastava S, Jalink M, Gyawali B. Text Messaging in Cancer-Supportive Care: A Systematic Review. Cancers (Basel) 2021;13:3542. https://doi.org/10.3390/cancers13143542.

[101] Caulfield RMH, Selman LE, Gibbins J, Forbes K, Chamberlain C. Enhanced supportive care in cancer centres: national cross-sectional survey. BMJ Support Palliat Care 2024:spcare-2023-004326. https://doi.org/10.1136/spcare-2023-004326.

[102] Villarreal-Garza C, Platas A, Miaja M, Mesa-Chavez F, Garcia-Garcia M, Fonseca A, et al. Patients’ satisfaction with a supportive care program for young breast cancer patients in Mexico: Joven &amp; Fuerte supports patients’ needs and eases their illness process. Supportive Care in Cancer 2020;28:4943–51. https://doi.org/10.1007/s00520-020-05334-y.

[103] Avancini A, Trestini I, Tregnago D, Belluomini L, Sposito M, Insolda J, et al. Willingness, preferences, barriers, and facilitators of a multimodal supportive care intervention including exercise, nutritional and psychological approach in patients with cancer: a cross-sectional study. J Cancer Res Clin Oncol 2023;149:3435–45. https://doi.org/10.1007/s00432-022-04232-6.

[104] Afiyanti Y, Setyowati, Milanti A, Young A. ‘Finally, I get to a climax’: the experiences of sexual relationships after a psychosexual intervention for Indonesian cervical cancer survivors and the husbands. J Psychosoc Oncol 2020;38:293–309. https://doi.org/10.1080/07347332.2020.1720052.

[105] Avery J, Schulte HK, Campbell KL, Bates A, McCune L, Howard AF. “What We Want Is More Access…”: Experiences of Supportive Cancer Care and Strategies for Advancement in a Canadian Provincial Cancer Care Organization. Current Oncology 2021;28:2227–38. https://doi.org/10.3390/curroncol28030205.

[106] Bankole AO, Burse NR, Crowder V, Chan Y-N, Hirschey R, Jung A, et al. “A strong reason why I enjoy coming to work”: Clinician acceptability of a palliative and supportive care intervention (PACT) for older adults with acute myeloid leukemia and their care partners. J Geriatr Oncol 2024;15:101740. https://doi.org/10.1016/j.jgo.2024.101740.

[107] Boltong A, Ledwick M, Babb K, Sutton C, Ugalde A. Exploring the rationale, experience and impact of using Cancer Information and Support (CIS) services: an international qualitative study. Supportive Care in Cancer 2017;25:1221–8. https://doi.org/10.1007/s00520-016-3513-7.

[108] Burton S, Miller L, Churcher C, Hanna L, Jones R, Hudson E, et al. Redesigning follow-up care for cancer patients. Cancer Nursing Practice 2013;12:12–7. https://doi.org/10.7748/cnp2013.07.12.6.12.e931.

[109] Cormie P, Turner B, Kaczmarek E, Drake D, Chambers SK. A Qualitative Exploration of the Experience of Men With Prostate Cancer Involved in Supervised Exercise Programs. Oncol Nurs Forum 2015;42:24–32. https://doi.org/10.1188/15.ONF.24-32.

[110] Evered J, Andersen L, Foxwell A, Iroegbu C, Whitney C. The impact and implications of virtual supportive cancer care during the COVID-19 pandemic: integrating patient and clinician perspectives. Supportive Care in Cancer 2022;30:9945–52. https://doi.org/10.1007/s00520-022-07393-9.

[111] Montiel C, Bedrossian N, Kramer A, Myre A, Piché A, McDonough MH, et al. Barriers and facilitators of supportive care access and use among men with cancer: a qualitative study. Journal of Cancer Survivorship 2025;19:306–18. https://doi.org/10.1007/s11764-023-01467-1.

[112] Ralph N, Chambers S, Laurie K, Oliffe J, Lazenby M, Dunn J. Nurse-Led Supportive Care Intervention for Men With Advanced Prostate Cancer: Healthcare Professionals’ Perspectives. Oncol Nurs Forum 2020;47:33–43. https://doi.org/10.1188/20.ONF.33-43.

[113] Regnier Denois V, Querre M, Chen L, Barrault M, Chauvin F. Inequalities and Barriers to the Use of Supportive Care Among Young Breast Cancer Survivors: a Qualitative Understanding. Journal of Cancer Education 2017;32:790–8. https://doi.org/10.1007/s13187-016-1087-1.

[114] Young J, Snowden A. A qualitative study on the perceived impact of using an integrated community‐based supportive cancer service. Eur J Cancer Care (Engl) 2019;28. https://doi.org/10.1111/ecc.13001.

[115] McLaughlin K, Hedden L, Pollock P, Higano C, Murphy RA. Assessing the nutritional needs of men with prostate cancer. Nutr J 2019;18:81. https://doi.org/10.1186/s12937-019-0506-7.
